# Association of Primary Care Consultation Patterns With Early Signs and Symptoms of Psychosis

**DOI:** 10.1001/jamanetworkopen.2018.5174

**Published:** 2018-11-30

**Authors:** Sarah A. Sullivan, William Hamilton, Kate Tilling, Theresa Redaniel, Paul Moran, Glyn Lewis

**Affiliations:** 1 Centre for Academic Primary Care, University of Bristol, Bristol, United Kingdom; 2 National Institute for Health Research Collaboration for Leadership in Applied Health Research and Care West, University Hospitals Bristol National Health Service Foundation Trust, University of Bristol, Bristol, United Kingdom; 3 Exeter Medical School, University of Exeter, Exeter, United Kingdom; 4 School of Social and Community Medicine, University of Bristol, Bristol, United Kingdom; 5 Centre for Academic Mental Health, University of Bristol, Bristol, United Kingdom; 6 Division of Psychiatry, University College London, London, United Kingdom

## Abstract

**Importance:**

Primary care is an important part of the care pathway for patients with psychosis; therefore, primary care physicians need to be able to accurately identify those at clinical high risk of psychosis. The difficulty of this task is increased because clinical high-risk symptoms are frequently nonspecific to psychosis.

**Objective:**

To determine whether the consultation patterns for a prespecified set of symptoms can be used to identify primary care patients who later developed a psychotic illness.

**Design, Setting, and Participants:**

This nested case-control study used primary care consultation data collected from 530 primary care practices in 13 UK regions from January 1, 2000, through September 30, 2009. Participants included 11 690 adults with a diagnosis of psychosis and 81 793 control participants who did not have a diagnosis of psychosis individually matched by age group, sex, and primary care practice. Data were analyzed from July 1, 2015, through June 2, 2017.

**Exposures:**

Prespecified symptoms selected from literature included attention-deficit/hyperactivity disorder–like symptoms, bizarre behavior, blunted affect, problems associated with cannabis, depressive symptoms, role functioning problems, social isolation, symptoms of mania, obsessive-compulsive disorder–like symptoms, disordered personal hygiene, sleep disturbance, problems associated with cigarette smoking, and suicidal behavior (including self-harm).

**Main Outcomes and Measures:**

Case (diagnosis of psychosis) or control (no diagnosis of psychosis) status. Conditional logistic regression was used to investigate the association between symptoms and case-control status in the 5 years before diagnosis. Positive predictive values (PPVs) were calculated using the Bayes theorem for symptoms stratified by age group and sex. Repeated-measures Poisson regression was used to investigate symptom consultation rate.

**Results:**

Of the total sample of 93 483 participants, 57.4% were female and 40.0% were older than 60 years (mean [SD] age, 51.34 [21.75] years). Twelve symptoms were associated with a later psychotic diagnosis (all prespecified symptoms except disordered personal hygiene). The strongest association was with suicidal behavior (odds ratio [OR], 19.06; 95% CI, 16.55-21.95). Positive predictive values were heterogeneous across age and sex. The highest PPVs were for suicidal behavior (33.0% in men 24 years or younger [95% CI, 24.2%-43.2%] and 19.6% in women aged 25-34 years [95% CI, 13.7%-27.2%]). Pairs of symptoms were associated with an increase in PPV. Consultation rates were higher in cases and increased 3 months before diagnosis.

**Conclusions and Relevance:**

Most of the preselected nonspecific symptoms were associated with a later psychotic diagnosis, particularly among young men consulting for suicidal behavior, especially if consulting with increasing frequency. These symptoms should alert physicians to patients who may benefit from a further assessment of psychotic symptoms.

## Key Points

**Question:**

Are primary care consultation patterns for prespecified nonspecific symptoms associated with a psychotic diagnosis?

**Findings:**

In this case-control study of 11 690 adults with psychosis and 81 793 matched controls without psychosis, 12 clinical high-risk symptoms, analyzed by sex and age group, were associated with a psychosis diagnosis, with suicidal behavior having the highest associated risk for psychosis. Pairs of symptoms were associated with an increase in the positive predictive value for a diagnosis of psychosis.

**Meaning:**

These findings are the first stage in the development and validation of a prognostic model of psychosis for primary care by providing candidate predictors and counter the generally held belief that individuals with emerging psychosis do not seek help from a medical professional.

## Teaser

This nested case-control study evaluates whether the consultation patterns for a prespecified set of symptoms can be used to identify patients attending primary care who later developed a psychotic illness.

## Introduction

The clinical and social outcomes of psychosis are often poor. Approximately 25% of people experience a relapse within the first 3 years of treatment,^1^ and residual symptoms are common.^2^ Many risk factors for a poor outcome (eg, sex or low socioeconomic status^3^) are difficult or impossible to modify. Consequently, interest has been increasing in the duration of untreated psychosis (ie, the time from the first psychotic symptom and receiving specialist treatment) because evidence suggests that duration of untreated psychosis is positively associated with poorer outcomes^4^ and is potentially modifiable.

The belief that duration of untreated psychosis is associated with poorer outcomes has influenced the structure of mental health services in the United Kingdom and internationally,^4^ resulting in provision of services to reduce duration of untreated psychosis and thereby improve outcomes. In the United Kingdom, these services have taken the form of Early Intervention for Psychosis Teams (EITs).^5^ The EITs accept referrals of those patients considered to be at clinical high risk (CHR) of psychosis as well as those with developed disorders. Prognostic tests (eg, Comprehensive Assessment of At-Risk Mental States^6^) are administered in EITs to assess the risk of future psychosis development. Such tests perform moderately in help-seeking populations but poorly in general populations,^7^ where psychosis incidence is much lower. Potentially, therefore, only a small proportion of those at risk of psychosis in the general population are referred to EITs.

Care pathways to specialist care for people with psychosis vary. In the United States, evidence suggests that primary care has a central role in psychiatric disorder.^8^ In the United Kingdom, most people (ie, 98%) are registered with primary care,^9^ which is the most common (ie, 50%-76%) route into specialist care, although 11% to 24% enter via routes such as the police^10^; however, a proportion of these persons likely have previously engaged with primary care. Some evidence suggests that a shorter duration of untreated psychosis is associated with more primary care visits before the diagnosis date^11^; primary care physicians are therefore an important part of the care pathway for people with psychosis. As a consequence, primary care physicians must recognize those at CHR to expedite referral to EITs for early treatment. The accuracy of diagnoses recorded on primary care electronic records has been found to be valid,^12,13^ but primary care physicians underidentify insidious CHR symptoms.^14^ This finding is problematic because CHR symptoms are frequently nonspecific and so may presage other health problems. Also, most primary care physicians see few new cases of psychosis each year and therefore have little opportunity to increase personal experience. Ideally, primary care physicians would have better information about the symptoms that could help them identify patients at CHR and who should undergo further assessment for psychotic symptoms.

The natural history of CHR symptoms of psychosis in the general population is poorly understood, and knowledge in this area is largely derived from studies of CHR samples,^15,16,17,18,19^ but in these studies patients were already receiving specialist mental health treatment. We are aware of 2 studies that have investigated CHR symptoms in general population samples.^20,21^ Both studies collected symptoms before diagnosis, but the number of outcomes was small, not allowing the discounting of alternative explanations for the findings. A variety of CHR symptoms have been reported as associated with a later diagnosis of psychosis, including unusual thought content, disorganized communication,^15^ sleep disturbance,^16^ anxiety, depression, feeling in need of treatment for an eating disorder,^20^ obsessive-compulsive disorder,^17^ social withdrawal,^21^ premorbid adjustment,^19^ and increasing use of cannabis.^22^

In this study we had 2 aims. First, we described the symptoms that are part of CHR. Second, we evaluated the clinical utility of the symptoms in terms of their association and positive predictive value (PPV). We hypothesized that prespecified CHR symptoms would be associated with later diagnosis and that these symptoms would have potential clinical utility.

## Methods

### Data Source

This case-control study follows the Strengthening the Reporting of Observational Studies in Epidemiology (STROBE) reporting guidelines. We used data from the General Practice Research Database now named the Clinical Practice Research Datalink (CPRD) (https://www.cprd.com). This computerized database of anonymized longitudinal primary care records has been maintained by the Medicines and Healthcare Products Regulatory Agency for nearly 30 years. At the time of analysis, the database covered approximately 3 million active patients (ie, approximately 5% of the UK population). The demographic distribution of patients on the database is similar to that of the UK as a whole. Participating primary care practices enter demographic, diagnostic, consultation, prescribing, and referral data using a preset library of 100 035 codes encompassing all facets of UK primary care. The CPRD is, in effect, an anonymized copy of these records. Validation studies^23^ report that the quality and completeness of data are high. Ethical approval was obtained from the Clinical Practice Research Datalink Independent Scientific Advisory Committee, which waived the need for informed consent for the use of publicly available data.

### Study Design and Sample

We conducted an individually matched, case-control study nested within the General Practice Research Database cohort. The total sample consisted of 93 483 people whose data were collected from 530 UK primary care practices. The cases included 11 690 people with an incident medical diagnosis of psychosis (including schizophrenia) documented from January 1, 2000, through December 31, 2009 (for code list eTable 1 in the Supplement). The index date of diagnosis was the first date that a diagnosis of psychosis was recorded. Cases had at least 5 years of up-to-standard follow-up recorded before the index date. The control group included patients in the data set without a psychosis code ever recorded on the database. Controls also had at least 5 years of up-to-standard follow-up recorded before the index date of the case. They were individually matched to cases by age group (5-year age bands), sex, and primary care practice. We requested 7 controls per case but accepted fewer if 7 matches were not found (n = 81 793). Exclusion criteria were a lack of 5 years of up-to-standard follow-up data for the 5 years before the index date.

### Primary Outcome

The primary outcome was a diagnosis of psychosis or otherwise (ie, case or control status). The date of diagnosis (index date) was identified as having the first recorded appropriate code.

### Explanatory Variables

Symptoms reported in primary care consultations for the 5 years before the index date constituted explanatory variables. Putative CHR symptoms were selected a priori, based on published literature.^24,25,26^ The symptoms were attention-deficit/hyperactivity disorder (ADHD)–like symptoms, bizarre behavior, blunted affect, problems with cannabis, depressive symptoms, role functioning problems, social isolation, mania, obsessive-compulsive disorder–like symptoms, disordered personal hygiene, sleep disturbance, problems with cigarette smoking, and suicidal behavior (including self-harm). A symptom library was compiled for each symptom using an established protocol^27^ (eTable 2 in the Supplement). As a check we used a negative exposure control^28^ by compiling a symptom library for sore throat, which has no theoretical association with a psychosis diagnosis. Covariates included actual age at entry to the study and time registered with primary care practice.

### Sample Size and Power

The number of cases and controls was predefined; therefore, we based our power calculation on an assumed potential number of 6700 cases with a diagnosis of psychosis in the General Practice Research Database from January 1, 2000, through September 30, 2009. We determined that this number would give us approximately 100% power to detect a difference in reporting of a rare symptom from 1% to 2% between case and control groups and the same power for a common symptom from 25% to 30% (2-tailed α set at 5%). Smaller differences would have little clinical meaning. By having 3 physicians in the study group, we ensured that all detected differences had clinical as well as statistical relevance.

### Statistical Analysis

Data were analyzed from July 1, 2015, through June 2, 2017. Initially we investigated the variability in PPVs within each symptom by sex and age group using a meta-analysis and *I*^2^ and Cochrane *Q* statistics. These results indicated whether we needed to investigate each symptom as a predictor within each sex and age group.

We investigated the association between each CHR symptom and a psychosis diagnosis using conditional logistic regression to account for the matching of cases and controls, with a diagnosis of psychosis or not as the binary outcome, using a significance level of *P* ≤ .001 and the sensitivity (true-positive divided by true-positive plus false-negative findings) and PPV (true-positive divided by true-positive plus false-positive findings) of each symptom.

Because of the case-control study design, we used the Bayes theorem to derive study PPVs and associated CIs. The theorem uses prior odds of psychosis in the population (eTable 3 in the Supplement). An initial literature search was conducted using the electronic library database Medline for published data on psychosis incidence. The search revealed a recent systematic review and meta-analysis^29^ that reported a psychosis incidence interaction of age and sex. Incidence rates were reported by age and sex group in 5-year age bands. From these data we derived probabilities (p) and then prior odds using p as p/(1 − p). The sex- and age group–specific incident proportion or cumulative incidence p was calculated using the fundamental association between cumulative incidence and incidence rates. Calculation of incident proportions was also accumulated across wider (10-year) age bands to fit the age categories for our analysis.

We did not calculate PPVs unless at least 10 cases consulted with the symptom, and 95% CIs were not calculated if 10 or fewer observations were found in any cell of the 2 × 2 table. We further investigated the PPV of pairs of CHR symptoms across age groups and sex and consultation rates for CHR symptoms before the index date using repeated-measures Poisson regression to investigate any change in the number of consultations for each symptom across time. The interval before the index date was categorized (>5 years, 4-5 years, 1-3 years, 7-12 months, 4-6 months, and ≤3 months), because we considered it would be of greater clinical utility than an association with a continuous time variable expressed as a β coefficient. The categories chosen were considered the most useful by the physicians in the study team. Consultation rates were compared between cases and controls, and each interval was compared with the reference category of more than 5 years. For the sensitivity analysis, we investigated the possibility of reverse causality by excluding symptoms that occurred less than 6 months and less than 1 year before diagnosis.

## Results

We obtained the study sample of 93 483 participants (42.6% male and 57.4% female; age, 51.34 [21.75] years; 40.0% of participants were older than 60 years) from 530 primary care practices in 13 UK regions. In total, 11 690 cases and 81 793 matched controls were identified (Table 1). Most participants were older than 19 years. The age spread was even across older bands with slightly fewer participants aged 61 to 70 years. Cases consulted their primary care physician a mean of 14 times more often than controls in the period before the index date.

**Table 1.** Characteristics of Cases With Psychosis and Matched Controls

Characteristic	Study Group	
	Cases (n = 11 690)	Controls (n = 81 793)^a^
Age, y, No. (%)		
<19	80 (0.7)	551 (0.7)
20-40	3041 (26.0)	20 612 (25.9)
41-60	3824 (32.7)	25 928 (32.5)
61-70	1371 (11.7)	9362 (11.7)
>70	3374 (28.9)	23 243 (29.2)
Male, No. (%)	5179 (44.3)	34 620 (43.4)
No. of consultations for any symptom in the 5 y before the index date, mean (SD)^b^	44.68 (29.97)	30.68 (24.53)

^a^ Data were missing for some controls.

^b^ *P* < .001, unpaired *t* test.

### Association Between CHR Symptoms and Psychosis Diagnosis

We found an association between all symptoms and psychosis diagnosis (Table 2), except disordered personal hygiene (odds ratio [OR], 2.60; 95% CI, 0.66-10.26; *P* = .17). The strongest association was with suicidal behavior (odds ratio [OR], 19.06; 95% CI, 16.55-21.95; *P* < .001). Consultation for sore throat was weakly associated with a psychosis diagnosis (OR, 1.09; 95% CI, 1.03-1.16; *P* = .003) (eTable 4 in the Supplement).

**Table 2.** Multivariable Conditional Logistic Regression of the Association Between Symptoms Recorded During Primary Care Consultations and a Diagnosis of Psychosis

Symptom	Study Group, No. (%)		OR (95% CI)^a^	*P* Value
	Cases (n = 11 690)	Controls (n = 81 793)		
Bizarre behavior	16 (0.1)	5 (0.01)	21.70 (7.94-59.28)	<.001
Suicidal behavior	762 (6.5)	326 (0.4)	19.06 (16.55-21.95)	<.001
Cannabis-associated problems	90 (0.8)	37 (0.04)	15.92 (11.23-22.58)	<.001
Depressive symptoms	7639 (65.4)	13 256 (16.2)	12.11 (11.53-12.72)	<.001
Blunted affect	17 (0.1)	16 (0.02)	7.69 (3.83-15.44)	<.001
ADHD-like symptoms	216 (1.8)	237 (0.3)	7.22 (5.96-8.74)	<.001
OCD-like symptoms	143 (1.2)	144 (0.2)	6.91 (5.50-8.69)	<.001
Social isolation	68 (0.6)	61 (0.1)	6.64 (5.05-8.74)	<.001
Role functioning problems	90 (0.8)	132 (0.2)	5.60 (4.39-7.15)	<.001
Symptoms of mania	2457 (21.0)	5122 (6.3)	4.66 (4.39-4.93)	<.001
Sleep disturbance	846 (7.2)	2424 (3.0)	3.22 (2.94-3.54)	<.001
Personal hygiene problems	3 (0.02)	9 (0.01)	2.60 (0.66-10.26)	.17
Smoking-associated problems^b^	3170 (27.1)	13 820 (16.9)	2.00 (1.90-2.10)	<.001

Abbreviations: ADHD, attention-deficit/hyperactivity disorder; OCD, obsessive-compulsive disorder; OR, odds ratio.

^a^ Adjusted for years registered with primary care practice and age at diagnosis.

^b^ Includes tobacco only.

### Sensitivity for Symptoms by Age Group and Sex

Sensitivity was generally low (see eFigure 1 in the Supplement for sensitivity statistics for prodromal symptoms by age group and sex). The highest sensitivities for both sexes were for depressive symptoms (0.70 for men and 0.85 for women aged 35-44 years), smoking-associated problems (0.35 for men aged 35-44 years and 0.40 for women aged 25-34 years), and mania symptoms (0.22 for men and 0.26 for women aged 45-54 years). For each of these symptoms, sensitivity was higher for women and highest in both sexes for those aged 25 to 34 years.

### PPV for Individual Symptoms by Age Group and Sex

**Figure 1.**
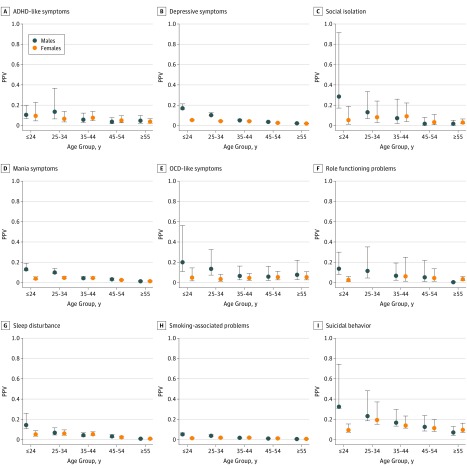
Positive Predictive Values (PPVs) per Prodromal Symptom by Age Group and Sex ADHD indicates attention-deficit/hyperactivity disorder; OCD, obsessive-compulsive disorder; data points, estimates; and error bars, 95% CI.

The meta-analysis confirmed variability of PPVs across age groups and sex (see eFigure 2 in the Supplement for forest plot of effect size with associated 95% CIs); therefore, PPV analyses were stratified. Table 3 and Figure 1 (PPVs for each prodromal symptom by age group and sex) show PPVs and 95% CIs where calculated. In general, all symptoms had moderate to high PPVs and 95% CIs that did not include null. The highest overall effect size (weighted by standard error) was for suicidal behavior (PPV, 33.0% [95% CI, 24.2%-43.2%] for men 24 years and younger; 19.6% [95% CI, 13.7%-27.2%] for women aged 25-34 years) and lowest for smoking-associated problems (PPV, 0.6% [95% CI, 0.6%-0.7%] for men 55 years and older). The greatest variability and highest PPVs across age and sex were for suicidal behavior (range of PPVs, 7.2% [95% CI, 4.5%-11.3%] for men 55 years and older to 33.0% [95% CI, 24.2%-43.2%] for men 24 years and younger) and the lowest variability and lowest PPVs were for smoking-associated problems (range of PPVs, 0.6% [95% CI, 0.6%-0.7%] for men 55 years and older to 5.4% [95% CI, 4.8%-6.1%] for men 24 years and younger).

**Table 3.** Univariable Analysis of Clinical High-Risk Symptoms^a^

Sex	Age Group, y	PPV (95% CI), %^b^	Likelihood Ratio (95% CI)
**ADHD-like Symptoms**			
Male	≤24	10.8 (6.9-16.6)	4.7 (2.9-7.8)
	25-34	13.9 (6.5-27.1)	7.9 (3.4-18.2)
	35-44	5.9 (3.1-10.8)	6.2 (3.2-12.0)
	45-54	3.9 (2.1-7.0)	5.7 (3.1-10.7)
	≥55	4.9 (2.6-8.8)	10.2 (5.4-19.1)
Female	≤24	9.7 (4.8-18.5)	10.6 (5.0-22.5)
	25-34	6.9 (3.9-12.1)	5.7 (3.1-10.5)
	35-44	7.8 (4.9-12.0)	6.9 (4.3-11.3)
	45-54	5.1 (3.0-8.6)	6.7 (3.8-11.6)
	≥55	4.1 (2.7-6.1)	6.0 (3.9-9.2)
**Cannabis Problems**			
Male	≤24	38.6 (25.9-53.1)	24.6 (13.7-44.2)
	25-34	24.5 (14.2-38.8)	15.9 (8.1-31.0)
	35-44	6.7 (2.5-12.4)	6.0 (2.5-14.0)
**Depressive Symptoms**			
Male	≤24	17.4 (15.8-19.2)	8.2 (7.3-9.2)
	25-34	10.4 (9.6-11.3)	5.7 (5.2-6.3)
	35-44	5.4 (5.0-5.8)	5.7 (5.2-6.1)
	45-54	3.6 (3.3-4.0)	5.4 (4.9-5.9)
	≥55	2.1 (2.0-2.3)	4.4 (4.1-4.7)
Female	≤24	5.2 (4.8-5.7)	5.4 (5.0-6.0)
	25-34	4.4 (4.2-4.7)	3.5 (3.3-3.7)
	35-44	4.1 (3.9-4.3)	3.5 (3.4-3.7)
	45-54	2.7 (2.6-2.8)	3.4 (3.3-3.6)
	≥55	2.0 (1.9-2.1)	2.9 (2.8-3.0)
**Role Functioning Problems**			
Male	≤24	14.1 (8.1-23.5)	6.4 (3.4-12.0)
	≥55	2.8 (1.7-4.4)	5.6 (3.5-9.2)
Female	≤24	2.8 (1.4-5.7)	2.9 (1.4-6.0)
	≥55	3.5 (2.2-5.4)	5.2 (3.3-8.1)
**Social Isolation**			
Male	≤24	29.1 (15.3-48.3)	16.0 (7.0-36.5)
	25-34	13.5 (6.8-25.2)	7.7 (3.6-16.5)
Female	35-44	9.0 (4.3-18.1)	8.2 (3.7-18.2)
	≥55	3.5 (2.0-5.8)	5.1 (2.9-8.8)
**Mania Symptoms**			
Male	≤24	13.5 (11.1-16.3)	6.1 (4.9-7.6)
	25-34	10.4 (9.3-13.8)	5.7 (4.7-6.9)
	35-44	4.6 (3.9-5.4)	4.7 (4.0-5.7)
	45-54	3.4 (2.9-4.1)	5.0 (4.2-6.1)
	≥55	1.4 (1.3-1.6)	2.9 (2.6-3.3)
Female	≤24	4.2 (3.3-5.3)	4.3 (3.4-5.5)
	25-34	4.9 (4.1-5.8)	3.9 (3.3-4.7)
	35-44	4.7 (4.1-5.3)	4.1 (3.6-4.6)
	45-54	2.8 (2.5-3.2)	3.6 (3.2-4.1)
	≥55	1.6 (1.4-1.7)	2.2 (2.1-2.4)
**OCD-like Symptoms**			
Male	≤24	20.6 (10.5-36.5)	10.0 (4.6-22.5)
	25-34	14.0 (7.5-24.8)	8.0 (4.0-16.1)
	35-44	6.7 (3.1-14.0)	7.2 (3.2-16.2)
	45-54	6.1 (2.5-13.8)	9.2 (3.7-22.7)
	≥55	7.9 (3.2-18.0)	17.0 (6.6-43.7)
Female	25-34	3.6 (1.8-7.3)	2.9 (1.4-6.0)
	35-44	4.8 (2.8-8.1)	4.1 (2.3-7.2)
	45-54	5.6 (3.1-9.9)	7.3 (3.9-13.7)
	≥55	5.5 (3.1-9.6)	8.3 (4.6-15.0)
**Sleep Disturbance**			
Male	≤24	14.7 (10.2-20.9)	6.7 (4.4-10.3)
	25-34	7.1 (4.8-10.3)	3.7 (2.5-5.6)
	35-44	4.5 (3.3-6.1)	4.6 (3.4-6.4)
	45-54	3.3 (2.4-4.5)	4.8 (3.5-6.7)
	≥55	1.0 (0.8-1.2)	2.0 (1.7-2.4)
Female	≤24	5.3 (3.6-7.9)	5.6 (3.7-8.4)
	25-34	6.2 (4.5-8.6)	5.1 (3.6-7.2)
	35-44	5.5 (4.3-7.0)	4.8 (3.7-6.2)
	45-54	2.4 (1.9-3.2)	3.1 (2.4-4.1)
	≥55	1.0 (0.9-1.1)	1.4 (1.3-1.6)
**Smoking-Associated Problems**			
Male	≤24	5.4 (4.8-6.1)	2.2 (2.0-2.5)
	25-34	3.9 (3.5-4.4)	2.0 (NR)
	35-44	1.9 (1.7-2.1)	1.9 (1.7-2.1)
	45-54	1.2 (1.0-1.3)	1.7 (1.5-1.9)
	≥55	0.6 (0.6-0.7)	1.3 (1.2-1.4)
Female	≤24	1.5 (1.3-1.7)	1.5 (1.8-2.2)
	25-34	2.0 (1.8-2.2)	1.5 (1.3-1.7)
	35-44	2.1 (1.9-2.3)	1.7 (1.6-1.9)
	45-54	1.3 (1.2-1.4)	1.6 (1.5-1.8)
	≥55	0.8 (0.8-0.9)	1.2 (1.1-1.3)
**Suicidal Behavior**			
Male	≤24	33.0 (24.2-43.2)	19.2 (12.5-29.7)
	25-34	23.4 (16.1-32.7)	15.0 (9.4-23.9)
	35-44	16.8 (12.2-22.9)	20.1 (13.7-29.4)
	45-54	12.7 (8.2-19.1)	20.6 (12.7-33.5)
	≥55	7.2 (4.5-11.3)	15.4 (9.4-25.4)
Female	≤24	9.8 (7.1-13.2)	10.7 (7.6-15.1)
	25-34	19.6 (13.7-27.2)	18.5 (12.1-28.3)
	35-44	14.3 (10.7-18.9)	13.7 (9.8-19.2)
	45-54	11.5 (7.9-16.3)	16.1 (10.7-24.2)
	≥55	9.9 (6.9-13.9)	15.5 (10.6-22.8)

Abbreviations: ADHD, attention-deficit/hyperactivity disorder; NR, not reported; OCD, obsessive-compulsive disorder; PPV, positive predictive value.

^a^ Analysis uses prior odds of psychosis dependent on age and sex.

^b^ Not calculated if fewer than 10 cases had this symptom (ie, bizarre behavior, blunted affect, cannabis problems for men older than 44 years and women of all ages, role functioning problems for men aged 25-54 years and women younger than 55 years, social isolation for men 35 years or older and women 34 years or younger and aged 45-54 years, OCD-like symptoms in women 24 years or younger, and personal hygiene for both sexes at all ages). See eTable 3 in the Supplement for prior odds used for each age group and sex.

Across most symptoms, the highest PPVs were for young men. Differences between PPVs for men and women in the youngest groups were largest for depressive symptoms (17.4% [95% CI, 15.8%-19.2%] for men vs 5.2% [95% CI, 4.8%-5.7%] for women), social isolation (29.1% [95% CI, 15.3%-48.3%] for men vs 9.0% [4.3%-18.1%] for women), mania symptoms (13.5% [95% CI, 11.1%-16.3%] for men vs 4.2% [95% CI, 3.3%-5.3%] for women), sleep disturbance (14.7% [95% CI, 10.2%-20.9%] for men vs 5.3% [95% CI, 3.6%-7.9%] for women), and suicidal behavior (33.0% [24.2%-43.2%] for men vs 9.8% [95% CI, 7.1%-13.2%] for women). However, for ADHD-like symptoms, PPVs for young men and women were similar (10.8% [95% CI, 6.9%-16.6%] for men and 9.7% [95% CI, 4.8%-18.5%] for women). In general, PPVs for all symptoms decreased with age. The PPVs for sore throat were universally small and were smallest for the oldest groups.

### PPVs for Symptom Pairs

We found a general pattern that symptom pairs increased the PPV of single symptoms (eTable 5 in the Supplement). For example, ADHD-like symptoms alone had a PPV of 11.6% (95% CI, 10.9%-15.7%), whereas with suicidal behavior the PPV increased to 29.5% (95% CI, 24.2%-72.3%). This change was particularly marked with suicidal behavior, which approximately tripled the PPVs of the accompanying symptom. The biggest increase in PPV was for mania and suicidal behavior (30.7% [95% CI, 24.7%-56.3%]) compared with mania alone (6.1% [95% CI, 6.3%-6.8%]).

### Consultation Rates Before Diagnosis

**Figure 2.**
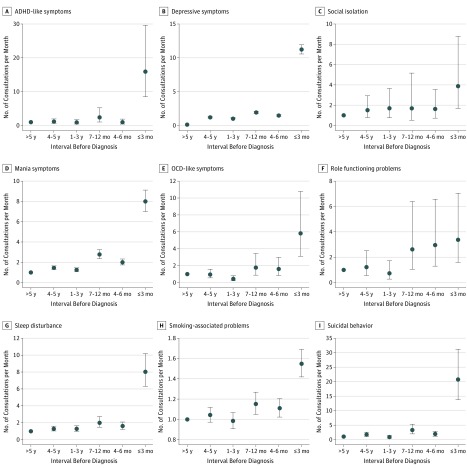
Consultation Rates for Prodromal Symptoms Before Diagnosis for Cases vs Controls Interval of longer than 5 years is the reference category. ADHD indicate attention-deficit/hyperactivity disorder; OCD, obsessive-compulsive disorder; data points, estimates; and error bars, 95% CI.

Every symptom reported by cases increased during the 5 years before the index date, compared with controls, although the timing of the increase differed. For ADHD-like, depressive, and mania symptoms, sleep disturbance, smoking-associated problems, and suicidal behavior, a sharp increase in consultations occurred in the final 3 months before the index date. For other symptoms there was a more gradual increase (Figure 2). Results of the sensitivity analysis were materially unchanged (eTable 6 in the Supplement).

## Discussion

This study is the first, to our knowledge, to prospectively describe the CHR state of psychosis in a general population sample. Our findings show strong associations between all symptoms except disordered personal hygiene and a psychosis diagnosis as well as more frequent consultations for symptoms within 5 years before diagnosis. The strong associations between symptoms that included diagnostic labels in their symptom libraries (ie, depressive and ADHD-like symptoms) suggest that part of the association may be due to misdiagnosis. However, a patient could also have a previous valid mental health diagnosis. An association has been reported^30^ for a previous nonpsychotic diagnosis and later psychotic diagnosis. We found evidence of variability in PPV estimates between symptoms and across age groups and sexes and that pairs of symptoms increased the PPV compared with single symptoms, particularly when suicidal behavior was added.

Consultation patterns over time for cases and controls differed. Not only did cases consult more frequently, but the rate of consultations per month increased as the diagnosis date approached. Our findings also suggest that people with emerging psychosis seek help, in contrast to the commonly held view that a lack of insight prevents this. We also found that, for certain symptoms, particularly of mood disturbance, consultation pattern differences were apparent 5 years before diagnosis.

These important findings represent a prospective description of the CHR, indicating the nature and development of the association between CHR symptoms and later diagnosis. Evidence is particularly convincing for young men consulting for suicidal behavior, obsessive-compulsive disorder–like symptoms, and social isolation, especially if consultations are increasingly frequent. These findings provide clinically useful information for primary care physicians who should consider that these symptoms reported by young men may be associated with psychosis, rather than other mental health disorders.

### Comparison With Existing Literature

Our findings support and extend those of other prospective general population studies. One study^20^ also found that anxiety and depression at ages 15 to 16 years were associated with a later psychosis diagnosis. A second study^21^ found that social withdrawal and difficulty in contact with others at age 14 years were associated with psychosis diagnosis in early adulthood. Our finding of increasing consultation rates is similar to that of a previous investigation into primary care consultation rates and suicide,^31^ which reported an increase in the 3 months before death.

### Strengths and Limitations

Our study represents an important improvement to existing evidence. The number of outcomes in previous studies^20,21^ was small (30 and 23, respectively), and therefore associations with rarer CHR symptoms may have been missed. Both studies also only investigated the association between adolescent CHR symptoms and later diagnosis, whereas our study covers a wider age range. No previous studies, to our knowledge, have used primary care data, which is important because 98% of the UK population is registered with a primary care physician, and these physicians are an important part of the treatment pathway for psychosis internationally. In the United Kingdom, primary care is the first point of contact for health care. In the United States and many other developed countries, patients are able to bypass primary care to access specialist services. However, we believe that our findings will be useful to any health care system that has some level of primary care services or where general physicians are often consulted.

The large primary care data source has enabled us to use previously recorded data and thereby avoid recall bias. The study design of a case-control study nested within a historical cohort is an important strength and has decreased the effects of physician-recording error, because accuracy is unlikely to be different in case and control groups. Evidence from Price et al^32^ suggests that a percentage of symptoms associated with some cancer diagnoses are recorded in free text, rather than preset codes, of the CPRD and that this was more prevalent in controls. If this suggestion is also true of psychosis, we may have overestimated ORs and PPVs.

One unexpected result was the small female preponderance of cases. No clear methodologic reason explains this, and it is unlikely to have unduly influenced the results. One possibility is that women are more likely to seek help. The association between primary care consultations for cannabis problems and a later psychosis diagnosis may be due to reporting bias (ie, a primary care physician may only ask about cannabis consumption if he or she suspects emerging psychosis).

Owing to the case-control structure of our research database, we were not able to investigate whether the prodromal symptoms used in our analyses were specifically indicative of psychosis, rather than other mental health disorders. This issue is important for the development of a clinical prediction model and should be further investigated using a database with a different structure in future studies with this aim.

Another possible limitation is unrecorded cases. Projects linking CPRD with Hospital Episode Statistics data report that diagnoses recording in CPRD data are incomplete.^33^ Also, delays may occur in making and recording diagnoses owing to reluctance to use a stigmatizing diagnosis and in the administrative task of diagnosis recording in primary care. If these unrecorded cases have also reported CHR symptoms, we may have underestimated the association.

A potential methodologic flaw is in the definition of symptom libraries. We probably did not produce an exhaustive list for each library, resulting in fewer classifications of CHR symptoms. This measurement error is unbiased and will result in an underestimate of associations.

We tested our methods with a negative exposure control group.^28^ Our finding of a weak association and low PPVs confirms validity of our method. The control group contains patients diagnosed with other mental health conditions. Nonspecific symptoms are more likely to be associated with mental health than physical conditions, which may have underestimated the true association.

Some cases may already have developed psychosis. In our sensitivity analysis we excluded all symptoms that were reported 6 months and 1 year before diagnosis, and the findings were not materially different. However, this timing issue may have affected our results.

A small proportion of cases may have experienced a very long CHR period of more than 5 years. In these cases, the first recorded symptom consultation may not be the actual first consultation. This CHR period is unlikely to have materially affected our findings, and previous evidence^34^ suggests that these patients are likely to be few.

Some of the ORs for the rarer symptoms, such as bizarre behavior, may have been overestimated owing to the very low prevalence of these symptoms. For instance, a change of 1 in the number of rarer symptoms could lead to a large change in ORs.

Any study using primary care data is affected by selection bias because some societal groups are less likely to be registered with a primary care physician and are also more likely to have a mental disorder. This bias may have resulted in an underestimate of association. In addition, our findings are not generalizable to people who do not consult primary care physicians before a psychosis diagnosis. In brief, most of the study limitations would result in an underestimate of associations; despite this, our estimates are relatively large.

## Conclusions

This information will help primary care physicians undertake the difficult task of determining which patients, consulting with nonspecific symptoms, are more likely to develop psychosis. Our methods could be used by primary care physicians to flag those patients who need further assessment and refine the population referred to specialist CHR services within EITs and have also detected candidate predictors for the development and validation of a prognostic model. Our findings may also reduce the number of those with psychosis who reach secondary care via routes such as emergency services, because some may have an identifiable pattern in prior primary care consultations. Our work indicates that some patients consult their primary care physician as long as 5 years before the diagnosis, providing a lengthy window of opportunity for early referral.

## Notes

Supplement. **eTable 1.** READ Codes Used for Diagnosis of Psychosis **eTable 2.** Methods and Search Terms Used to Compile Symptom Libraries **eTable 3.** Method for Deriving PPVs and Associated CIs Using Bayes Theorem **eTable 4.** Association Between Consultation for Sore Throat and Diagnosis of Psychosis **eTable 5.** PPVs and 95% CIs of Pairs of Prodromal Symptoms **eTable 6.** Sensitivity Analyses: Association Between Prodromal Symptoms and Case-Control Status **eFigure 1.** Sensitivity Statistics for Prodromal Symptoms by Age Group and Sex **eFigure 2.** Forest Plot of Effect Sizes With Associated 95% CIs

## References

[1] Addington D, Addington MD, Patten S Relapse rates in an early psychosis treatment service. Acta Psychiatr Scand. 2007;115(2):-. doi:10.1111/j.1600-0447.2006.00879.x 17244176

[2] Harrison G, Hopper K, Craig T, Recovery from psychotic illness: a 15- and 25-year international follow-up study. Br J Psychiatry. 2001;178:506-517. doi:10.1192/bjp.178.6.506 11388966

[3] Simonsen E, Friis S, Haahr U, Clinical epidemiologic first-episode psychosis: 1-year outcome and predictors. Acta Psychiatr Scand. 2007;116(1):54-61. doi:10.1111/j.1600-0447.2006.00942.x 17559601

[4] Marshall M, Lewis S, Lockwood A, Drake R, Jones P, Croudace T Association between duration of untreated psychosis and outcome in cohorts of first-episode patients: a systematic review. Arch Gen Psychiatry. 2005;62(9):975-983. doi:10.1001/archpsyc.62.9.975 16143729

[5] The National Archives The NHS plan: a plan for investment, a plan for reform. http://webarchive.nationalarchives.gov.uk/+/http://www.dh.gov.uk/en/Publicationsandstatistics/Publications/PublicationsPolicyAndGuidance/DH_4002960. Published July 1, 2000. Accessed June 1, 2017.

[6] Yung AR, Yuen HP, McGorry PD, Mapping the onset of psychosis: the Comprehensive Assessment of At-Risk Mental States. Aust N Z J Psychiatry. 2005;39(11-12):964-971. doi:10.1080/j.1440-1614.2005.01714.x 16343296

[7] Fusar-Poli P, Cappucciati M, Rutigliano G, At risk or not at risk? a meta-analysis of the prognostic accuracy of psychometric interviews for psychosis prediction. World Psychiatry. 2015;14(3):322-332. doi:10.1002/wps.20250 26407788 PMC4592655

[8] Abed Faghri NM, Boisvert CM, Faghri S Understanding the expanding role of primary care physicians (PCPs) to primary psychiatric care physicians (PPCPs): enhancing the assessment and treatment of psychiatric conditions. Ment Health Fam Med. 2010;7(1):17-25. 22477919 PMC2925161

[9] Herrett E, Gallagher AM, Bhaskaran K, Data resource profile: Clinical Practice Research Datalink (CPRD). Int J Epidemiol. 2015;44(3):827-836. doi:10.1093/ije/dyv098 26050254 PMC4521131

[10] Anderson KK, Fuhrer R, Malla AK The pathways to mental health care of first-episode psychosis patients: a systematic review. Psychol Med. 2010;40(10):1585-1597. doi:10.1017/S0033291710000371 20236571

[11] Skeate A, Jackson C, Birchwood M, Jones C Duration of untreated psychosis and pathways to care in first-episode psychosis: investigation of help-seeking behaviour in primary care. Br J Psychiatry Suppl. 2002;43:s73-s77. doi:10.1192/bjp.181.43.s73 12271804

[12] Nazareth I, King M, Haines A, Rangel L, Myers S Accuracy of diagnosis of psychosis on general practice computer system. BMJ. 1993;307(6895):32-34. doi:10.1136/bmj.307.6895.32 8343670 PMC1678461

[13] Herrett E, Thomas SL, Schoonen WM, Smeeth L, Hall AJ Validation and validity of diagnoses in the General Practice Research Database: a systematic review. Br J Clin Pharmacol. 2010;69(1):4-14. doi:10.1111/j.1365-2125.2009.03537.x 20078607 PMC2805870

[14] Platz C, Umbricht DS, Cattapan-Ludewig K, Help-seeking pathways in early psychosis. Soc Psychiatry Psychiatr Epidemiol. 2006;41(12):967-974. doi:10.1007/s00127-006-0117-4 17036265 PMC1764202

[15] Addington J, Liu L, Buchy L, North American Prodrome Longitudinal Study (NAPLS 2): the prodromal symptoms. J Nerv Ment Dis. 2015;203(5):328-335. doi:10.1097/NMD.0000000000000290 25919383 PMC4417745

[16] Lunsford-Avery JR, LeBourgeois MK, Gupta T, Mittal VA Actigraphic-measured sleep disturbance predicts increased positive symptoms in adolescents at ultra high-risk for psychosis: a longitudinal study. Schizophr Res. 2015;164(1-3):15-20. doi:10.1016/j.schres.2015.03.013 25818627 PMC4409558

[17] Zink M, Schirmbeck F, Rausch F, Obsessive-compulsive symptoms in at-risk mental states for psychosis: associations with clinical impairment and cognitive function. Acta Psychiatr Scand. 2014;130(3):214-226. doi:10.1111/acps.12258 24571191

[18] Salokangas RKR, Heinimaa M, From T, ; EPOS group Short-term functional outcome and premorbid adjustment in clinical high-risk patients: results of the EPOS project. Eur Psychiatry. 2014;29(6):371-380. doi:10.1016/j.eurpsy.2013.10.003 24315804

[19] Fusar-Poli P, Nelson B, Valmaggia L, Yung AR, McGuire PK Comorbid depressive and anxiety disorders in 509 individuals with an at-risk mental state: impact on psychopathology and transition to psychosis. Schizophr Bull. 2014;40(1):120-131. doi:10.1093/schbul/sbs136 23180756 PMC3885287

[20] Bratlien U, Øie M, Haug E, Self-reported symptoms and health service use in adolescence in persons who later develop psychotic disorders: a prospective case-control study. Early Interv Psychiatry. 2015;9(3):221-227. doi:10.1111/eip.12102 24224904

[21] Mäki P, Koskela S, Murray GK, Difficulty in making contact with others and social withdrawal as early signs of psychosis in adolescents—the Northern Finland Birth Cohort 1986. Eur Psychiatry. 2014;29(6):345-351. doi:10.1016/j.eurpsy.2013.11.003 24440523

[22] Kelley ME, Wan CR, Broussard B, Marijuana use in the immediate 5-year premorbid period is associated with increased risk of onset of schizophrenia and related psychotic disorders. Schizophr Res. 2016;171(1-3):62-67. doi:10.1016/j.schres.2016.01.015 26785806 PMC4929616

[23] Walley T, Mantgani A The UK General Practice Research Database. Lancet. 1997;350(9084):1097-1099. doi:10.1016/S0140-6736(97)04248-7 10213569

[24] Cornblatt BA, Lencz T, Smith CW, Correll CU, Auther AM, Nakayama E The schizophrenia prodrome revisited: a neurodevelopmental perspective. Schizophr Bull. 2003;29(4):633-651. doi:10.1093/oxfordjournals.schbul.a007036 14989404

[25] McGorry PD, McKenzie D, Jackson HJ, Waddell F, Curry C Can we improve the diagnostic efficiency and predictive power of prodromal symptoms for schizophrenia? Schizophr Res. 2000;42(2):91-100. doi:10.1016/S0920-9964(99)00125-5 10742647

[26] Klosterkotter J, Hellmich M, Schultze-Lutter F Is it possible to diagnose schizophrenia at the start of the initial prodromal phase prior to the first psychotic manifestation? [in German]. Fortschr Neurol Psychiatr. 2000;68:S13-S21. 10907608

[27] Hamilton W The CAPER studies: five case-control studies aimed at identifying and quantifying the risk of cancer in symptomatic primary care patients. Br J Cancer. 2009;101(suppl 2):S80-S86. doi:10.1038/sj.bjc.6605396 19956169 PMC2790706

[28] Lipsitch M, Tchetgen Tchetgen E, Cohen T Negative controls: a tool for detecting confounding and bias in observational studies. Epidemiology. 2010;21(3):383-388. doi:10.1097/EDE.0b013e3181d61eeb 20335814 PMC3053408

[29] Kirkbride JB, Errazuriz A, Croudace TJ, Incidence of schizophrenia and other psychoses in England, 1950-2009: a systematic review and meta-analyses. PLoS One. 2012;7(3):e31660. doi:10.1371/journal.pone.0031660 22457710 PMC3310436

[30] Lewis G, David AS, Malmberg A, Allebeck P Non-psychotic psychiatric disorder and subsequent risk of schizophrenia: cohort study. Br J Psychiatry. 2000;177:416-420. doi:10.1192/bjp.177.5.416 11059994

[31] Appleby L, Amos T, Doyle U, Tomenson B, Woodman M General practitioners and young suicides: a preventive role for primary care. Br J Psychiatry. 1996;168(3):330-333. doi:10.1192/bjp.168.3.330 8833687

[32] Price SJ, Stapley SA, Shephard E, Barraclough K, Hamilton WT Is omission of free text records a possible source of data loss and bias in Clinical Practice Research Datalink studies? a case-control study. BMJ Open. 2016;6(5):e011664. doi:10.1136/bmjopen-2016-011664 27178981 PMC4874123

[33] Herrett E, Shah AD, Boggon R, Completeness and diagnostic validity of recording acute myocardial infarction events in primary care, hospital care, disease registry, and national mortality records: cohort study. BMJ. 2013;346(f2350):f2350. doi:10.1136/bmj.f2350 23692896 PMC3898411

[34] Birchwood M, Lester H, McCarthy L, The UK National Evaluation of the Development and Impact of Early Intervention Services (the National EDEN studies): study rationale, design and baseline characteristics. Early Interv Psychiatry. 2014;8(1):59-67. doi:10.1111/eip.12007 23347742

