# Supplementary material for: Association of Primary Care Consultation Patterns With Early Signs and Symptoms of Psychosis
Source: JAMA Netw Open. 2018 Nov 30;1(7):e185174. doi: 10.1001/jamanetworkopen.2018.5174 (PMC6324409; doi:10.1001/jamanetworkopen.2018.5174)
Supplement: Supplement. — eTable 1. READ Codes Used for Diagnosis of Psychosis eTable 2. Methods and Search Terms Used to Compile Symptom Libraries eTable 3. Method for Deriving PPVs and Associated CIs Using Bayes Theorem eTable 4. Association Between Consultation for Sore Throat and Diagnosis of Psychosis eTable 5. PPVs and 95% CIs of Pairs of Prodromal Symptoms eTable 6. Sensitivity Analyses: Association Between Prodromal Symptoms and Case-Control Status eFigure 1. Sensitivity Statistics for Prodromal Symptoms by Age Group and Sex eFigure 2. Forest Plot of Effect Sizes With Associated 95% CIs [file jamanetwopen-1-e185174-s001.pdf]

Supplementary Online Content

Sullivan SA, Hamilton W, Tilling K, Redaniel T, Moran P, Lewis G. Association of primary care consultation patterns with early signs and symptoms of psychosis. *JAMA Netw Open*. 2018;1(7):e185174. doi:10.1001/jamanetworkopen.2018.5174

eTable 1. READ Codes Used for Diagnosis of Psychosis

eTable 2. Methods and Search Terms Used to Compile Symptom Libraries

eTable 3. Method for Deriving PPVs and Associated CIs Using Bayes Theorem

eTable 4. Association Between Consultation for Sore Throat and Diagnosis of Psychosis

eTable 5. PPVs and 95% CIs of Pairs of Prodromal Symptoms

eTable 6. Sensitivity Analyses: Association Between Prodromal Symptoms and Case-Control Status

eFigure 1. Sensitivity Statistics for Prodromal Symptoms by Age Group and Sex

eFigure 2. Forest Plot of Effect Sizes With Associated 95% CIs

This supplementary material has been provided by the authors to give readers additional information about their work.

© 2018 Sullivan SA et al. *JAMA Network Open.*

Supplementary Material

eTable 1. READ Codes Used for Diagnosis of Psychosis

medcode	readcode	readterm	flag
23963	ZV11111	[V]Personal history of manic-depressive psychosis	Inclusion
22080	ZV11112	[V]Personal history of manic-depressive psychosis	Inclusion
22104	ZV11000	[V]Personal history of schizophrenia	Inclusion
47619	Eu02z12	[X] Presenile psychosis NOS	Inclusion
27759	Eu02z16	[X] Senile dementia, depressed or paranoid type	Inclusion
27935	Eu02z15	[X] Senile psychosis NOS	Inclusion
24387	Eu04.13	[X]Acute / subacute infective psychosis	Inclusion
34168	Eu23z00	[X]Acute and transient psychotic disorder, unspecified	Inclusion
25019	Eu23.00	[X]Acute and transient psychotic disorders	Inclusion
11778	Eu23200	[X]Acute schizophrenia-like psychotic disorder	Inclusion
31633	Eu3z.11	[X]Affective psychosis NOS	Inclusion
30162	Eu10513	[X]Alcoholic paranoia	Inclusion
17607	Eu10514	[X]Alcoholic psychosis NOS	Inclusion
24062	Eu84111	[X]Atypical childhood psychosis	Inclusion
91547	Eu20311	[X]Atypical schizophrenia	Inclusion
28277	Eu31200	[X]Bipolar affect disorder cur epi manic with psychotic symp	Inclusion
54387	Eu21.12	[X]Borderline schizophrenia	Inclusion
31707	Eu23z11	[X]Brief reactive psychosis NOS	Inclusion
61501	Eu20200	[X]Catatonic schizophrenia	Inclusion
31738	Eu2y.11	[X]Chronic hallucinatory psychosis	Inclusion
24107	Eu20511	[X]Chronic undifferentiated schizophrenia	Inclusion
21455	Eu23012	[X]Cycloid psychosis	Inclusion
26143	Eu23112	[X]Cycloid psychosis with symptoms of schizophrenia	Inclusion
62222	Eu84312	[X]Disintegrative psychosis	Inclusion
53985	Eu20111	[X]Disorganised schizophrenia	Inclusion
23731	Eu33311	[X]Endogenous depression with psychotic symptoms	Inclusion
6709	Eu05y11	[X]Epileptic psychosis NOS	Inclusion
43405	Eu20100	[X]Hebephrenic schizophrenia	Inclusion
28168	Eu44.14	[X]Hysterical psychosis	Inclusion
47230	Eu24.12	[X]Induced paranoid disorder	Inclusion
11973	Eu24.13	[X]Induced psychotic disorder	Inclusion
61304	Eu84013	[X]Infantile psychosis	Inclusion
50248	Eu22y12	[X]Involutional paranoid state	Inclusion
11670	Eu10611	[X]Korsakov's psychosis, alcohol induced	Inclusion
57993	Eu03.11	[X]Korsakov's psychosis, nonalcoholic	Inclusion
64993	Eu21.13	[X]Latent schizophrenia	Inclusion
98417	Eu32800	[X]Major depression, severe with psychotic symptoms	Inclusion
37102	Eu30211	[X]Mania with mood-congruent psychotic symptoms	Inclusion
48632	Eu30212	[X]Mania with mood-incongruent psychotic symptoms	Inclusion
21065	Eu30200	[X]Mania with psychotic symptoms	Inclusion
28677	Eu33312	[X]Manic-depress psychosis,depressed type+psychotic symptoms	Inclusion
6710	Eu31.12	[X]Manic-depressive psychosis	Inclusion
38429	Eu12500	[X]Mental & behav dis due to cannabinoids: psychotic disordr	Inclusion

© 2018 Sullivan SA et al. *JAMA Network Open.*

54983	Eu16500	[X]Mental & behav dis due to hallucinogens: psychotic disord	Inclusion
69138	Eu13500	[X]Mental & behav dis due to seds/hypntcs: psychotic disordr	Inclusion
12353	Eu10500	[X]Mental & behav dis due to use alcohol: psychotic disorder	Inclusion
49565	Eu14500	[X]Mental & behav dis due to use cocaine: psychotic disorder	Inclusion
50964	Eu11500	[X]Mental & behav dis due to use opioids: psychotic disorder	Inclusion
98618	Eu18500	[X]Mental & behav dis due to vol solvents: psychotic disordr	Inclusion
49879	Eu15500	[X]Mental/behav dis oth stims inc caffeine: psychotic dis	Inclusion
37580	Eu25212	[X]Mixed schizophrenic and affective psychosis	Inclusion
21986	Eu05200	[X]Organic delusional [schizophrenia-like] disorder	Inclusion
21985	Eu0z.11	[X]Organic psychosis NOS	Inclusion
44503	Eu23y00	[X]Other acute and transient psychotic disorders	Inclusion
44307	Eu23300	[X]Other acute predominantly delusional psychotic disorders	Inclusion
30985	Eu2y.00	[X]Other nonorganic psychotic disorders	Inclusion
49420	Eu20y00	[X]Other schizophrenia	Inclusion
4843	Eu22015	[X]Paranoia	Inclusion
55236	Eu22y13	[X]Paranoia querulans	Inclusion
24009	Eu05211	[X]Paranoid organic state	Inclusion
21338	Eu60000	[X]Paranoid personality disorder	Inclusion
2113	Eu22011	[X]Paranoid psychosis	Inclusion
16764	Eu20000	[X]Paranoid schizophrenia	Inclusion
11172	Eu22012	[X]Paranoid state	Inclusion
50060	Eu20011	[X]Paraphrenic schizophrenia	Inclusion
62449	Eu21.14	[X]Prepsychotic schizophrenia	Inclusion
40386	Eu21.15	[X]Prodromal schizophrenia	Inclusion
49852	Eu21.16	[X]Pseudoneurotic schizophrenia	Inclusion
71250	Eu21.17	[X]Pseudopsychopathic schizophrenia	Inclusion
27770	Eu23312	[X]Psychogenic paranoid psychosis	Inclusion
694	Eu2z.11	[X]Psychosis NOS	Inclusion
17614	Eu53111	[X]Puerperal psychosis NOS	Inclusion
29651	Eu23z12	[X]Reactive psychosis	Inclusion
32941	Eu33313	[X]Recurr severe episodes/major depression+psychotic symptom	Inclusion
31757	Eu33314	[X]Recurr severe episodes/psychogenic depressive psychosis	Inclusion
16861	Eu33315	[X]Recurrent severe episodes of psychotic depression	Inclusion
37764	Eu33316	[X]Recurrent severe episodes/reactive depressive psychosis	Inclusion
64264	Eu20500	[X]Residual schizophrenia	Inclusion
33410	Eu25z11	[X]Schizoaffective psychosis NOS	Inclusion
35274	Eu25111	[X]Schizoaffective psychosis, depressive type	Inclusion
16905	Eu25011	[X]Schizoaffective psychosis, manic type	Inclusion
34236	Eu20.00	[X]Schizophrenia	Inclusion
17281	Eu2..00	[X]Schizophrenia, schizotypal and delusional disorders	Inclusion
34966	Eu20z00	[X]Schizophrenia, unspecified	Inclusion
31877	Eu05212	[X]Schizophrenia-like psychosis in epilepsy	Inclusion
41022	Eu25112	[X]Schizophreniform psychosis, depressive type	Inclusion
51903	Eu25012	[X]Schizophreniform psychosis, manic type	Inclusion
48687	Eu60014	[X]Sensitive paranoid personality disorder	Inclusion
12099	Eu32300	[X]Severe depressive episode with psychotic symptoms	Inclusion
35848	Eu20600	[X]Simple schizophrenia	Inclusion
24117	Eu32311	[X]Single episode of major depression and psychotic symptoms	Inclusion

© 2018 Sullivan SA et al. *JAMA Network Open.*

52678	Eu32312	[X]Single episode of psychogenic depressive psychosis	Inclusion
24112	Eu32313	[X]Single episode of psychotic depression	Inclusion
28863	Eu32314	[X]Single episode of reactive depressive psychosis	Inclusion
53848	Eu84314	[X]Symbiotic psychosis	Inclusion
8766	Eu0z.12	[X]Symptomatic psychosis NOS	Inclusion
60013	Eu20300	[X]Undifferentiated schizophrenia	Inclusion
11244	Eu2z.00	[X]Unspecified nonorganic psychosis	Inclusion
97919	E101400	Acute exacerbation of chronic hebephrenic schizophrenia	Inclusion
53032	E103400	Acute exacerbation of chronic paranoid schizophrenia	Inclusion
63478	E107400	Acute exacerbation of chronic schizo-affective schizophrenia	Inclusion
44498	E100400	Acute exacerbation of chronic schizophrenia	Inclusion
51322	E103300	Acute exacerbation of subchronic paranoid schizophrenia	Inclusion
57666	E100300	Acute exacerbation of subchronic schizophrenia	Inclusion
58866	E107300	Acute exacerbation subchronic schizo-affective schizophrenia	Inclusion
29937	E131.00	Acute hysterical psychosis	Inclusion
15053	E133.00	Acute paranoid reaction	Inclusion
30404	E015.00	Alcoholic paranoia	Inclusion
67651	E01z.00	Alcoholic psychosis NOS	Inclusion
55467	E004200	Arteriosclerotic dementia with paranoia	Inclusion
33338	E10y000	Atypical schizophrenia	Inclusion
55829	E114400	Bipolar affect disord, currently manic,severe with psychosis	Inclusion
63701	E115400	Bipolar affect disord, now depressed, severe with psychosis	Inclusion
69155	E14y100	Borderline psychosis of childhood	Inclusion
23538	E13y100	Brief reactive psychosis	Inclusion
25546	E102.00	Catatonic schizophrenia	Inclusion
63867	E102z00	Catatonic schizophrenia NOS	Inclusion
92994	E10y.11	Cenesthopathic schizophrenia	Inclusion
52849	E14z.00	Child psychosis NOS	Inclusion
37395	E14z.11	Childhood schizophrenia NOS	Inclusion
94299	E105200	Chronic latent schizophrenia	Inclusion
39625	E04z.00	Chronic organic psychosis NOS	Inclusion
3890	E121.00	Chronic paranoid psychosis	Inclusion
31362	E103200	Chronic paranoid schizophrenia	Inclusion
43800	E107200	Chronic schizo-affective schizophrenia	Inclusion
99070	E10y100	Coenesthopathic schizophrenia	Inclusion
99000	E107.11	Cyclic schizophrenia	Inclusion
1915	1BH..00	Delusions	Inclusion
54464	ZR5..00	Delusions-symptoms-states inventory	Inclusion
56143	E141.00	Disintegrative psychosis	Inclusion
26002	E02z.00	Drug psychosis NOS	Inclusion
26481	E021z00	Drug-induced paranoia or hallucinatory state NOS	Inclusion
45997	E021.00	Drug-induced paranoia or hallucinatory states	Inclusion
12628	E021000	Drug-induced paranoid state	Inclusion
32875	1BH1.00	Grandiose delusions	Inclusion
12777	146H.00	H/O: psychosis	Exclusion
6325	1464	H/O: schizophrenia	Exclusion
30619	E101.00	Hebephrenic schizophrenia	Inclusion
67768	E101500	Hebephrenic schizophrenia in remission	Inclusion

© 2018 Sullivan SA et al. *JAMA Network Open.*

48054	E101z00	Hebephrenic schizophrenia NOS	Inclusion
23835	E040.11	Korsakoff's non-alcoholic psychosis	Inclusion
4500	E011000	Korsakov's alcoholic psychosis	Inclusion
11106	E011100	Korsakov's alcoholic psychosis with peripheral neuritis	Inclusion
66410	E105.00	Latent schizophrenia	Inclusion
96883	E105500	Latent schizophrenia in remission	Inclusion
54195	E116400	Mixed bipolar affective disorder, severe, with psychosis	Inclusion
9686	E2...00	Neurotic, personality and other nonpsychotic disorders	Inclusion
14965	E13z.00	Nonorganic psychosis NOS	Inclusion
22188	E1z..00	Non-organic psychosis NOS	Inclusion
60509	E2Az.00	Nonpsychotic mental disorder post-organic brain damage NOS	Inclusion
49509	E2A..00	Nonpsychotic mental disorders following organic brain damage	Inclusion
12472	225E.00	O/E - paranoid delusions	Inclusion
7233	E0...00	Organic psychotic conditions	Inclusion
33425	E11zz00	Other affective psychosis NOS	Inclusion
33670	E01y.00	Other alcoholic psychosis	Inclusion
68111	E01yz00	Other alcoholic psychosis NOS	Inclusion
31589	E12y.00	Other paranoid states	Inclusion
31455	E12yz00	Other paranoid states NOS	Inclusion
39062	E10y.00	Other schizophrenia	Inclusion
49761	E10yz00	Other schizophrenia NOS	Inclusion
66766	E12y000	Paranoia querulans	Inclusion
22643	1BH3.00	Paranoid ideation	Inclusion
5652	E210.00	Paranoid personality disorder	Inclusion
12771	E12z.00	Paranoid psychosis NOS	Inclusion
1494	E103.00	Paranoid schizophrenia	Inclusion
36172	E103500	Paranoid schizophrenia in remission	Inclusion
9281	E103z00	Paranoid schizophrenia NOS	Inclusion
4261	E12..00	Paranoid states	Inclusion
51494	E00y.11	Presbyophrenic psychosis	Inclusion
30032	E001200	Presenile dementia with paranoia	Inclusion
24345	E134.00	Psychogenic paranoid psychosis	Inclusion
4390	285..11	Psychotic condition, insight present	Inclusion
3636	E13z.11	Psychotic episode NOS	Inclusion
17770	E130.11	Psychotic reactive depression	Inclusion
8478	E130.00	Reactive depressive psychosis	Inclusion
24171	E113400	Recurrent major depressive episodes, severe, with psychosis	Inclusion
32295	E111400	Recurrent manic episodes, severe, with psychosis	Inclusion
38063	E106.00	Residual schizophrenia	Inclusion
2117	E107.00	Schizo-affective schizophrenia	Inclusion
10575	E107z00	Schizo-affective schizophrenia NOS	Inclusion
8407	E10z.00	Schizophrenia NOS	Inclusion
73295	E100.11	Schizophrenia simplex	Inclusion
62680	E123.00	Shared paranoid disorder	Inclusion
14743	E120.00	Simple paranoid state	Inclusion
32222	E100.00	Simple schizophrenia	Inclusion
53625	E100z00	Simple schizophrenia NOS	Inclusion
32159	E112400	Single major depressive episode, severe, with psychosis	Inclusion

© 2018 Sullivan SA et al. *JAMA Network Open.*

50218	E110400	Single manic episode, severe, with psychosis	Inclusion
99199	E102100	Subchronic catatonic schizophrenia	Inclusion
61098	E107100	Subchronic schizo-affective schizophrenia	Inclusion
23616	E100100	Subchronic schizophrenia	Inclusion
68326	E117400	Unspecified bipolar affective disorder,severe with psychosis	Inclusion
58716	E102000	Unspecified catatonic schizophrenia	Inclusion
66506	E101000	Unspecified hebephrenic schizophrenia	Inclusion
33383	E103000	Unspecified paranoid schizophrenia	Inclusion
2114	E03y300	Unspecified puerperal psychosis	Inclusion
58862	E107000	Unspecified schizo-affective schizophrenia	Inclusion
15733	E100000	Unspecified schizophrenia	Inclusion

© 2018 Sullivan SA et al. *JAMA Network Open.*

eTable 2. Methods and Search Terms Used to Compile Symptom Libraries

ADHD-like symptoms		
String search terms		
"Hyperactive"	"Concentration"	"Attention"
Search Results=4	Search Results=8	Search Results=18
[X]Attention deficit hyperactivity disorder	Poor concentration	Child attention deficit disorder
Attention deficit with hyperactivity	Reduced concentration	[X]Disturbance of activity and attention
Hyperactive behaviour	Lack of concentration	[X]Attention deficit hyperactivity disorder
Attention deficit without hyperactivity	Ability to concentrate	Attention deficit with hyperactivity
	Reduced concentration span	Child attention deficit disorder NOS
	Short concentration span	Short attention span
	Unable to concentrate	[X]Attention deficit disorder
	Ability to sustain concentration	ADD - Attention deficit disorder
		[X]Attention deficit disorder
		O/E - sensory inattention
		Short attention span
		Attention deficit disorder
		Attention deficit without hyperactivity
		Disorders of attention and motor control
		Deficits in attention motor control and perception
		Attention to direct verbal communication
		Reduced attention span
		[X]Deficits in attention, motor control and perception

Bizarre behaviour	
String search terms	
"Bizarre"	"Inappropriate"
Search Results=3	Search Results=3

© 2018 Sullivan SA et al. *JAMA Network Open.*

[D]Bizarre personal appearance	Inappropriate shouting
O/E - bizarre appearance	[V]Inappropriate diet and eating habits
Conversation content bizarre	Conversation content inappropriate

**Blunted Affect**
String search term
“Blunted Affect”
Search Results=1
Blunted Affect

**Problems with cannabis use**
String search term
“Cannabis”
Search Results=26
Nondependent cannabis abuse
Cannabis type drug dependence
[X]Mental and behavioural disorders due to use cannabinoids
[X]Drug addiction - cannabis
Cannabis dependence, unspecified
Nondependent cannabis abuse, episodic
Nondependent cannabis abuse NOS
Prolonged high dose use of cannabis
Cannabinosis
Cannabis drug dependence NOS
Adverse reaction to cannabis
[X]Mental & behav dis due cannabinoids: acute intoxication
[X]Mental & behav dis due to cannabinoids: psychotic disordr
Nondependent cannabis abuse, continuous
Nondependent cannabis abuse, unspecified

© 2018 Sullivan SA et al. *JAMA Network Open.*

Cannabis dependence, continuous
Cannabis dependence in remission
[X]Mental and behav dis due to use cannabinoids: harmful use
Cannabis dependence, episodic
Nondependent cannabis abuse in remission
[X]Mental and behav dis due to cannabinoids: dependence synd
[X]Mnt/bh dis due cannabinds: resid & late-onset psychot dis
[X]Men/behav dis due to use cannabinoids: oth men/behav disd
[X]Ment/behav dis due use cannabinoids: unsp ment/behav disd
[X]Mental and behav dis due cannabinoids: withdrawal state
[X]Mental and behav dis due to use cannabinoids: amnesic syn

**Depressive symptoms**		
String search terms		
"Anxiety"	"Depression"	"Mood"
Search Results=27	Search Results=128	Search Results=62
Anxiety states	Depressive disorder NEC	[X]Behavioural/emotional disords onset childhood/adolescence
[X]Anxiety neurosis	[X]Depression NOS	Rebound mood swings
Chronic anxiety	Endogenous depression	[X]Mood - affective disorders
H/O: anxiety state	Anxiety with depression	Mood swings
Anxiety state NOS	Agitated depression	Low mood
Recurrent anxiety	Neurotic depression reactive type	Depressed mood
Generalised anxiety disorder	[X]Manic-depressive illness	Disturbance of emotion specific to childhood and adolescence
[X]Other anxiety disorders	Brief depressive reaction	Elevated mood
Anxiety state unspecified	Depressed	Manic mood
[X]Panic disorder [episodic paroxysmal anxiety]	Depressive psychoses	[X]Atypical childhood psychosis
[X]Phobic anxiety disorders	Puerperal depression	Atypical childhood psychoses

© 2018 Sullivan SA et al. *JAMA Network Open.*

Phobic anxiety	[X]Depressive episode, unspecified	[X]Phobic anxiety disorder of childhood
[X]Generalized anxiety disorder	[X]Depressive disorder NOS	Unhappiness of childhood or adolescence
[X]Mixed anxiety and depressive disorder	[X]Recurrent depressive disorder	Sad mood
FH: Anxiety state	Chronic depression	O/E - fearful mood
[X]Persistant anxiety depression	[X]Depressive episode	Childhood and adolescence disturbance of unhappiness
[X]Anxiety disorder, unspecified	Bipolar affective disorder, currently depressed	[X]Other mood affective disorders
[X]Other specified anxiety disorders	C/O - feeling depressed	[X]Other specified mood affective disorders
[X]Anxiety NOS	Agitated depression	[X]Social anxiety disorder of childhood
[X]Other phobic anxiety disorders	[X] Reactive depression NOS	[X]Other recurrent mood affective disorders
[X]Anxiety hysteria	Recurrent depression	Profile of mood states, bipolar
Disturbance of anxiety and fearfulness childhood/adolescent	Endogenous depression first episode	Disturbance of anxiety and fearfulness childhood/adolescent
[X]Phobic anxiety disorder, unspecified	[X]Manic-depressive psychosis	Disturbance of emotion specific to childhood and adolescence
Disturbance anxiety and fearfulness childhood/adolescent NOS	[X]Other depressive episodes	Disturbance anxiety and fearfulness childhood/adolescent NOS
[X]Anxiety reaction	Endogenous depression - recurrent	Childhood and adolescent overanxiousness disturbance
[X]Other mixed anxiety disorders	Endogenous depression first episode	[X]Unspecified mood affective disorder
[X]Anxiety state	Single major depressive episode NOS	[X]Mania with mood-congruent psychotic symptoms
	[X]Single episode of reactive depression	Childhood schizophrenia NOS
	[X]Neurotic depression	Other childhood and adolescent emotional problems NOS
	[X]Mild anxiety depression	Childhood and adolescent relationship problem
	Reactive depressive psychosis	[X]Persistent mood affective disorder, unspecified
	[X]Depressive neurosis	Childhood and adolescent disturbance with shyness
	[X]Recurrent episodes of depressive reaction	[X]Persistent mood affective disorders
	[X]Recurrent episodes of reactive depression	Psychoses with origin in childhood
	[X]Single episode of depressive reaction	Childhood and adolescent disturbance with sensitivity

© 2018 Sullivan SA et al. *JAMA Network Open.*

	FH: Depression	Childhood and adolescent emotion disorder NOS
	Masked depression	[X]Emotional disorders with onset specific to childhood
	[X]Moderate depressive episode	[X]Disorder social funct onset specific childhood/adolesc
	[X]Severe depressive episode without psychotic symptoms	[X]Unspec behav emotion disorder onst usual childhood adoles
	Symptoms of depression	[X]Childhood disorder of social functioning, unspecified
	Depressed mood	Dysphoric mood
	[X]Depressive personality disorder	[X]Mania with mood-incongruent psychotic symptoms
	Depressive symptoms	Other childhood and adolescent emotional problems
	Depressive personality disorder	[X]Childhood emotional disorder, unspecified
	Single major depressive episode	[X]Other persistent mood affective disorders
	[X]Mild depression	[X]Other single mood affective disorders
	[X]Atypical depression	[X]Childhood behavioural disorder NOS
	[X]Schizoaffective disorder, depressive type	Childhood and adolescent fearfulness disturbance
	[X]Major depression, recurrent without psychotic symptoms	Childhood and adolescent disturbance with elective mutism
	[X]Endogenous depression without psychotic symptoms	[X]Ot spc behav emotion disorder onst usual childhood adoles
	H/O: manic depressive disorder	[X]Schizoid disorder of childhood
	Unspecified manic-depressive psychoses	Childhood and adolescent sensitivity disturbance NOS
	[X]Mild depressive episode	[X]Childhood overanxious disorder
	[X]Mixed anxiety and depressive disorder	Childhood and adolescent relationship problem NOS
	[X]Severe depressive episode with psychotic symptoms	[X]Other childhood emotional disorders
	Manic-depressive - now depressed	[X]Childhood identity disorder
	[X]Postnatal depression NOS	[V]Other negative life events in childhood
	Recurrent major depressive episodes, moderate	Other childhood psychoses NOS
	Recurrent major depressive episode	[X]Other childhood disintegrative disorder

© 2018 Sullivan SA et al. *JAMA Network Open.*

	Single major depressive episode, moderate	Borderline psychosis of childhood
	Single major depressive episode, severe, without psychosis	Other childhood psychoses
	[X]Persistant anxiety depression	Ecstatic mood
	Bipolar affective disorder, currently depressed, unspecified	
	Single major depressive episode, mild	
	[X]Bipolar affect disorder cur epi mild or moderate depressn	
	Prolonged depressive reaction	
	[X]Recurrent severe episodes of psychotic depression	
	Manic-depressive - now manic	
	Psychotic reactive depression	
	[X]Single episode of psychogenic depression	
	[X]Recurrent brief depressive episodes	
	[X]Recurrent episodes of psychogenic depression	
	[X]Post-schizophrenic depression	
	[V]Personal history of manic-depressive psychosis	
	[X]Recurrent depressive disorder, currently in remission	
	[X]Single episode major depression w'out psychotic symptoms	
	[X]Bipol aff disord, curr epis sev depress, no psychot symp	
	[X]Endogenous depression with psychotic symptoms	
	[V]Personal history of manic-depressive psychosis	
	[X]Single episode of psychotic depression	
	[X]Single episode of major depression and psychotic symptoms	
	Recurrent major depressive episodes, severe, with psychosis	
	Recurrent major depressive episode NOS	
	Recurrent major depressive episodes, severe, no psychosis	
	Atypical depressive disorder	
	Bipolar affective disorder, currently depressed, moderate	
	[X]Prolonged single episode of reactive depression	
	[X]Manic-depress psychosis,depressed type+psychotic symptoms	

© 2018 Sullivan SA et al. *JAMA Network Open.*

	[X]Seasonal depressive disorder	
	[X]Single episode of reactive depressive psychosis	
	Recurrent major depressive episodes, mild	
	[X]Manic-depress psychosis,depressd,no psychotic symptoms	
	[X]Recurrent depressive disorder, current episode moderate	
	[X]Recurrent depressive disorder, current episode mild	
	[X]Recurr severe episodes/psychogenic depressive psychosis	
	Single major depressive episode, severe, with psychosis	
	[X]Recurr severe episodes/major depression+psychotic symptom	
	Other and unspecified manic-depressive psychoses NOS	
	[X]Recurr depress disorder cur epi severe without psyc sympt	
	Single major depressive episode, unspecified	
	[X]Schizoaffective psychosis, depressive type	
	Bipolar affect disord, now depressed, severe, no psychosis	
	Recurrent major depressive episodes, unspecified	
	Bipolar affective disorder, currently depressed, mild	
	Brief depressive reaction NOS	
	[X]Monopolar depression NOS	
	Bipolar affective disorder, currently depressed, NOS	
	[X]Recurrent severe episodes/reactive depressive psychosis	
	[X]Schizophreniform psychosis, depressive type	
	[X]Single episode agitated depressn w'out psychotic symptoms	
	[X]Recurrent depressive disorder, unspecified	
	Drug-induced depressive state	
	[X]Recurrent depress disorder cur epi severe with psyc symp	
	[X]Other recurrent depressive disorders	
	[X]Single episode of psychogenic depressive psychosis	
	Recurrent major depressive episodes,partial/unspec remission	
	[X]Single episode of masked depression NOS	

© 2018 Sullivan SA et al. *JAMA Network Open.*

	[X]Single episode vital depression w'out psychotic symptoms	
	Other and unspecified manic-depressive psychoses	
	Bipolar affect disord, now depressed, severe with psychosis	
	[X]Mainc-depressive reaction	
	Other mixed manic-depressive psychoses	
	Bipolar affect disord, now depressed, part/unspec remission	
	[X]Vital depression, recurrent without psychotic symptoms	
	[X]Major depression, moderately severe	
	[X]Major depression, mild	
	[X]Major depression, severe without psychotic symptoms	
	[X]Major depression, severe with psychotic symptoms	

**Role functioning problems**			
String Search Terms			
"Absenteeism"	"Exam fail"	"Miss school"	"Unemployment"
Search Results=2	Search Results=2	Search Results=24	Search Results=5
Absenteeism	Failed exams	School problem	Unemployed
Absenteeism or truancy	[V]Failed examinations	School difficulties	Chronic unemployment
		School refusal	[V]Problems related to employment and unemployment
		School attendance poor	Recently unemployed
		Bullied at school	[V]Unemployment
		Expelled from school	
		[X]Truancy from school	
		Behavioural problems at school	
		[V]Underachievement in school	
		Temporary exclusion from school	
		School attendance	
		Forced to leave school	

© 2018 Sullivan SA et al. *JAMA Network Open.*

		Deterioration in school performance	
		Intentional non-attendance at school	
		Suspended from school	
		Excluded from school	
		Permanent exclusion from school	
		Left school free will	
		Excluded from school permanently	
		Bullied at school	
		School difficulties	
		Excluded from school temporarily	
		Victim of bullying when not in school	
		Parental withholding from school	
		Excluded from school temporarily	

**Social isolation**
String Search Term
"Social"
Search Results=39
Aggressive unsocial conduct disorder
[X]Socialized conduct disorder
Social problem
Social isolation
Social adjustment problem
[X]Social phobias
Social withdrawal
Social phobia, fear of eating in public
Social phobic disorders
Social phobia, fear of public washing

© 2018 Sullivan SA et al. *JAMA Network Open.*

Nonaggressive unsocial conduct disorder NOS
[V]Problems related to certain psychosocial circumstances
[V]Social maladjustment
Social maladjustment
Unsocial childhood truancy
Aggressive unsocial conduct disorder NOS
[V]Problems related to social environment
[X]Unsocialised aggressive disorder
[X]Social anxiety disorder of childhood
[V]Social role conflict, not elsewhere classified
[X]Dissocial personality disorder
Social phobia, fear of public speaking
Observations relating to complex and social behaviours
[X]Unsocialized conduct disorder
[X]Other childhood disorders of social functioning
Socialised conduct disorder NOS
[X]Social neurosis
Socialised childhood truancy
Socialised conduct disorder
Social outcast
[V]Observation for adolescent antisocial behaviour
[X]Disorder social funct onset specific childhood/adolesc
[X]Childhood disorder of social functioning, unspecified
Nonaggressive unsocial conduct disorder
[V]Social exclusion and rejection
[V]Observation for dyssocial behaviour
[X]Other problems related to social environment
[X]Inadequate social skills, not elsewhere classified
[D]Social skills development delay

© 2018 Sullivan SA et al. *JAMA Network Open.*

[X]Conduct disorder associated with emotional disorder

**Mania-like symptoms**			
String search term			
“Mania”	“Anxiety”	“Hyperactive”	“Sleep”
Search Results=4	Search Results=33	Search Results=4	Search Results=37
[X]Hypomania	Anxiety states	[X]Attention deficit hyperactivity disorder	[D]Sleep disturbance, unspecified
[X]Mania NOS	Anxiety with depression	Attention deficit with hyperactivity	Sleep disorders
[X]Mania with psychotic symptoms	[X]Anxiety neurosis	Hyperactive behaviour	Disorders of initiating and maintaining sleep
[X]Mania with mood-incongruent psychotic symptoms	Chronic anxiety	Attention deficit without hyperactivity	Poor sleep pattern
	H/O: anxiety state		Non-organic sleep disorders
	Anxiety state NOS		[D]Sleep disturbances
	Recurrent anxiety		[X]Sleep terrors
	Generalised anxiety disorder		[D]Sleep dysfunction NOS
	[X]Other anxiety disorders		[D]Sleep rhythm irregular
	Separation anxiety disorder		Unspecified non-organic sleep disorder
	Anxiety state unspecified		[D]Sleep rhythm inversion
	[X]Mild anxiety depression		Sleep drunkenness
	[X]Panic disorder [episodic paroxysmal anxiety]		Restless sleep
	[X]Phobic anxiety disorders		[X]Emotional sleep disorder NOS
	Phobic anxiety		Cannot sleep - insomnia
	[X]Generalized anxiety disorder		[X]Nonorganic sleep disorder, unspecified
	[X]Mixed anxiety and depressive disorder		[X]Nonorganic disorder of the sleep-wake schedule
	FH: Anxiety state		[X]Nonorganic sleep disorders
	[X]Persistant anxiety depression		Excessive sleep
	[X]Anxiety disorder, unspecified		Insomnia due to nonorganic sleep disorder
	[X]Other specified anxiety disorders		Non-organic sleep disorder NOS

© 2018 Sullivan SA et al. *JAMA Network Open.*

	[X]Phobic anxiety disorder of childhood		[D]Sleep rhythm problems
	[X]Anxiety NOS		Short-sleeper
	[X]Other phobic anxiety disorders		Inversion of sleep rhythm
	[X]Anxiety hysteria		Repeated rapid eye movement sleep interruptions
	[X]Social anxiety disorder of childhood		[D]Sleep dysfunction with arousal disturbance
	Disturbance of anxiety and fearfulness childhood/adolescent		Delayed onset of sleep
	[X]Phobic anxiety disorder, unspecified		Other non-organic sleep disorder
	Disturbance anxiety and fearfulness childhood/adolescent NOS		Repetitive intrusions of sleep
	[X]Anxiety reaction		[V]Personal history of unhealthy sleep-wake schedule
	[X]Other mixed anxiety disorders		[X]Other sleep disorders
	[X]Anxiety state		[D]Sleep dysfunction with sleep stage disturbance
			Other sleep stage or arousal dysfunction
			[D]Sleep-wake rhythm non-24-hour cycle
			Sleeping pattern
			Light sleep
			[X]Other nonorganic sleep disorders

**Obsessive compulsive disorder symptoms**
String search term
“Obsessive compulsive”
Search results=14
Obsessional personality
Obsessional neurosis
Obsessive-compulsive disorders
[X]Obsessive - compulsive disorder
Obsessive-compulsive disorder NOS
[X]Obsessive-compulsive personality disorder

© 2018 Sullivan SA et al. *JAMA Network Open.*

[X]Mixed obsessional thoughts and acts
Obsessional thoughts
[X]Predominantly obsessional thoughts or ruminations
[X]Obsessive-compulsive neurosis
[X]Predominantly compulsive acts [obsessional rituals]
[X]Obsessional personality disorder
[X]Obsessive-compulsive disorder, unspecified
[X]Other obsessive-compulsive disorders

**Poor personal hygiene**
String search term
“Hygiene”
Search Results=10
[V]Personal history of poor personal hygiene
[D]Very low level of personal hygiene
Neglect of personal hygiene
Does not perform personal hygiene activity
Ability to perform personal hygiene activity
Difficulty performing personal hygiene activity
Personal hygiene disability
Does not maintain standard of personal hygiene
Unable to maintain standard of personal hygiene
Difficulty maintaining standard of personal hygiene
Poor personal hygiene

**Sleep disturbance**
String search term “Sleep”
Search results=38

© 2018 Sullivan SA et al. *JAMA Network Open.*

[D]Sleep disturbance, unspecified
Sleep disorders
Disorders of initiating and maintaining sleep
Poor sleep pattern
Non-organic sleep disorders
[D]Sleep disturbances
[X]Sleep terrors
Disorders of the sleep-wake schedule
[D]Sleep dysfunction NOS
[D]Sleep rhythm irregular
Unspecified non-organic sleep disorder
[D]Sleep rhythm inversion
Sleep drunkenness
Restless sleep
[X]Emotional sleep disorder NOS
Cannot sleep - insomnia
[X]Nonorganic sleep disorder, unspecified
[X]Nonorganic disorder of the sleep-wake schedule
[X]Nonorganic sleep disorders
Excessive sleep
Insomnia due to nonorganic sleep disorder
Non-organic sleep disorder NOS
[D]Sleep rhythm problems
Short-sleeper
Inversion of sleep rhythm
Repeated rapid eye movement sleep interruptions
[D]Sleep dysfunction with arousal disturbance
Delayed onset of sleep
Other non-organic sleep disorder

© 2018 Sullivan SA et al. *JAMA Network Open.*

Repetitive intrusions of sleep
[V]Personal history of unhealthy sleep-wake schedule
[X]Other sleep disorders
[D]Sleep dysfunction with sleep stage disturbance
Other sleep stage or arousal dysfunction
[D]Sleep-wake rhythm non-24-hour cycle
Sleeping pattern
Light sleep
[X]Other nonorganic sleep disorders

**Cigarette smoking**
String Search term “Smoking”
Search Results=18
Smoking cessation advice
Advice on smoking
Smoking cessation milestones
Referral to stop-smoking clinic
Trying to give up smoking
Smoking restarted
Smoking started
Keeps trying to stop smoking
Smoking reduced
Referral to smoking cessation advisor
Thinking about stopping smoking
Not interested in stopping smoking
Ready to stop smoking
Negotiated date for cessation of smoking
Refuses stop smoking monitor

© 2018 Sullivan SA et al. *JAMA Network Open.*

Smoking status at 52 weeks
Smoking restarted
Reason for restarting smoking

**Suicidal behavior**	
Search term	Search term
“self-harm”	“suicidal”
Search results=65	Search results=68
H/O: deliberate self harm	Suicidal ideation
Deliberate self-harm	Suicide + selfinflicted poisoning by drug or medicine NOS
Self-harm	Attempted suicide
Intent of deliberate self harm with detailed plans	Para-suicide
Thoughts of deliberate self harm	[X]Suicide
[X]Intentional self-harm	Suicidal plans
At risk of DSH - deliberate self harm	Suicide risk
Harmful thoughts	Suicide + selfinflicted inj oth mean hang/strangle/suffocate
[V]Personal history of self-harm	[X]Para-suicide
[X]Intentional self harm by smoke, fire and flames	Suicidal - symptom
[X]Intentional self harm by sharp object	H/O: attempted suicide
[X]Intentional self harm by sharp object occurrence at home	Suicide and selfinflicted injury by hanging
[X]Intent self harm by hanging strangulation / suffocation	Suicide + selfinflicted poisoning by analgesic/antipyretic
[X]Intentional self harm by unspecified means	Suicide and selfinflicted injury by cutting
[X]Intent self harm by smoke fire/flames occ unspecif place	Suicidal
[X]Intentional self harm by other specified means	Suicide + selfinflicted poisoning by barbiturates
[X]Intentional self harm by drowning and submersion	[X]Attempted suicide
[X]Intent self harm by jumping / lying before moving object	Suicide and self harm
[X]Intentional self harm by unspecif means occurrn at home	Suicide and selfinflicted injury
[X]Intent self harm by hangng strangul/suffoct unspecif plce	Suicide + selfinflicted poisoning by other drugs/medicines

© 2018 Sullivan SA et al. *JAMA Network Open.*

[X]Intent self harm by other/unspecified firearm discharge	Suicide + selfinflicted injury by hang/strangulate/suffocate
[X]Sequel intentn self-harm assault+event of undeterm intent	Suicide and selfinflicted injury by jumping from high place
[X]Intentional self harm by jumping from a high place	Suicide and selfinflicted injury by stabbing
Plans for deliberate self harm without intent	Suicide + selfinflicted poisoning tranquilliser/psychotropic
[X]Intent self harm by rifle shotgun/larger firearm disch	Suicide + selfinflicted poisoning by corrosive/caustic subst
[X]Intentional self harm by sharp object occ unspecif place	Suicide and selfinflicted injury by shotgun
[X]Intent self harm by sharp object occ resident instit'n	Suicide + selfinflicted poisoning by solid/liquid substances
[X]Intent self harm by blunt object occ sports/athlet area	Suicide and selfinflicted injury by drowning
[X]Intent self harm by hanging strangulat/suffocat occ home	Suicide + selfinflicted poisoning by oth sedatives/hypnotics
[X]Intent self harm by jump from high place occ street/h'way	High suicide risk
[X]Int slf hrm rifl s'gun/lrg frarm dis sch/ins/pub adm area	Suicide and selfinflicted injury by scald
[X]Intentional self harm by steam hot vapours / hot objects	Suicide and selfinflicted injury by cutting and stabbing
[X]Intentional self harm by crashing of motor vehicle	Moderate suicide risk
[X]Intent self harm by drowning/submersn occ resid instit'n	Suicide and selfinflicted injury NOS
[X]Intent self harm by blunt object occ resident instit'n	Suicide+selfinflicted injury-jump from oth manmade structure
[X]Intent self harm by unspecif means occ at unspecif place	Suicide and selfinflicted injury by burns or fire
[X]Intentional self harm by blunt object	Suicide and selfinflicted injury by other means
[X]Int self harm jump/lying befr mov obje occ street/highway	Suicide + selfinflicted poisoning by motor veh exhaust gas
[X]Intent self harm by smoke fire/flame occ street/highway	Suicide and selfinflicted poisoning by other utility gas
[X]Intent self harm by hangng strangult/suffoct resid instit	Suicide and selfinflicted poisoning by other carbon monoxide
Suicide and selfinflicted injury by firearms and explosives	Suicide + selfinflicted injury by suffocation by plastic bag
[X]Intent self harm by steam hot vapour/hot obj occ at home	Suicide + selfinflicted poisoning by agricultural chemical
[X]Intentionl self harm by oth specif means occurrn at home	Suicide and selfinflicted injury by hunting rifle
[X]Int self harm rifl s'gun/lrg frarm disch occ resid instit	Suicide+selfinflicted injury-jump from high place NOS
[X]Intentional self harm by blunt object occurrence at home	Suicide + selfinflicted injury-jumping before moving object
[X]Intent self harm by oth specif means occ unspecif place	Suicide + selfinflicted inj by hang/strangle/suffocate NOS
[X]Intent self harm by jumping from high place occ at home	Suicide+selfinflicted injury-jump from natural sites
[X]Intention self harm by smoke fire/flames occurrn at home	Suicide+selfinflicted injury-jump from residential premises
[X]Intention self harm by sharp object occ oth specif place	Suicide + selfinflicted poisoning by other gases and vapours

© 2018 Sullivan SA et al. *JAMA Network Open.*

[X]Intent self harm by steam hot vapour/obj occ unspec place	Suicide and selfinflicted injury by crashing motor vehicle
[X]Intentional self harm by explosive material	Suicide and selfinflicted injury by other firearm
[X]Int self harm jump/lying bef mov obje occ oth specif plce	Suicide and selfinflicted injury by cutting and stabbing NOS
[X]Intent self harm by oth specif means occ resid instit'n	Suicide and selfinflicted injury by other specified means
[X]Intention self harm by sharp object occ street/highway	Suicide and self inflicted injury by Amylobarbitone
[X]Intent self harm by hangng strangul/suffoct oth spec plce	Suicide + selfinflicted poisoning by solid/liquid subst NOS
[X]Int self harm jump/lying befr mov obje occ resid instit'n	Suicide and selfinflicted injury by firearms and explosives
[X]Int self harm by jump from high place occ unspecif place	Suicide + selfinflicted poisoning by gas via pipeline
[X]Intent self harm by drown/submersn occ oth specif place	Suicide + selfinflicted poisoning by gases in domestic use
[X]Int self harm by jump from high place indust/constr area	Suicide + selfinflicted injury-jump/lie before moving object
[X]Intent self harm oth/unspecif firearm disch occ at home	Suicide and selfinflicted injury by electrocution
[X]Intent self harm by unspecif means occ oth specif place	Suicide + selfinflicted poisoning by liquified petrol gas
[X]Intent self harm by unspec mean occ sch/ins/pub adm area	Suicide and selfinflicted injury by other means NOS
[X]Intent self harm by drown/submersn occ unspecified place	Suicide + selfinflicted poisoning by gases and vapours NOS
[X]Inten slf harm hang strang/suffc sch oth ins/pub adm area	Suicide + selfinflicted poisoning by domestic gases NOS
[X]Int self harm by jump from high place occ oth specif plce	Suicide and self inflicted injury by Phenobarbitone
	Suicide and selfinflicted injury caustic subst, excl poison
	Suicide and selfinflicted injury by extremes of cold
	Suicide and self inflicted injury by Barbitone

Initially, the research group compiled a list of possible descriptions for each symptom by collating words plausibly used by primary-care clinicians during their recording of consultations i.e. for depressive symptoms words such as sadness, hopelessness, helplessness, anxiety and mood were used in addition to specific terms for depression. Word (string) searches were then carried out in the GPRD Medical Dictionary browser (version 13.1) using wild-card searches. The search results were copied into an excel spreadsheet and individually checked for relevance. All relevant symptoms were compiled into a symptom library for each symptom. Some symptom libraries could therefore include both a diagnosis and words used to describe a symptom i.e. a depression diagnosis and “feeling sad” as a description. Other symptom libraries– such as social isolation – did not, by definition, include a diagnosis.

Key: [X] – diagnosis ICD10; O/E – on examination; [D] – working diagnosis; [V] - supplementary factors influencing health status or contact with health services other than for illness; H/O – history of; FH – family history; C/O – complaining of; NOS – not otherwise specified.

© 2018 Sullivan SA et al. *JAMA Network Open.*

eTable 3. Method for Deriving PPVs and Associated CIs Using Bayes Theorem

Prior odds of of psychosis for each gender and age group obtained from published literature on incidence rates[1]

Gender	Age group (years)	Odds of psychosis onset
Males	≤24	0.025
	25-34	0.02
	35-44	0.01
	45-54	0.007
	≥55	0.005
Females	≤24	0.01
	25-34	0.013
	35-44	0.012
	45-54	0.008
	≥55	0.007

The published incidence rates[1] for different ages and genders were used to derive probabilities using the equation 1-exp(-lamda*time). Prior odds (see Table below) were then derived from the probabilities, using the equation p/(1-p) as prior odds for the derivation of the study PPV and associated confidence intervals for the predictive value of each prodromal symptom *within* gender and age of diagnosis (age groups ≤24 years, 25 to 34 years, 35 to 44 years, 45 to 54 years and ≥ 55 years) . The product of the prior odds (external to the study obtained from published evidence[1]) and the likelihood ratio (probability of psychosis in those with the symptom/probability of psychosis in those without the symptom) were used to derive posterior odds. PPVs and confidence intervals were derived from the posterior odds (posterior odds=prior odds*likelihood ratio; PPV=posterior odds/(1+posterior odds)).

1. Kirkbride J, Errazuriz A, Croudace T, et al. Incidence of schizophrenia and other psychoses in England 1950 - 2009: A systematic review and meta-analyses. *Plos One* 2012; **7**(3): 1-21.

© 2018 Sullivan SA et al. *JAMA Network Open.*

eTable 4. Association Between Consultation for Sore Throat and Diagnosis of Psychosis

	Unadjusted OR	95% CI	p
Sore throat	1.09	1.03, 1.16	0.003

Positive predictive values for consultation for sore throat and diagnosis of psychosis by age group and gender

Symptom	Gender	Age group (years)	PPV 95% CI	LR 95% CI
Sore throat	M	<=24	2.34 (2.02, 2.71)	0.93 (0.80, 1.08)
		25-34	2.00 (1.65, 2.42)	1.00 (0.82, 1.22)
		35-44	1.06 (0.88, 1.29)	1.06 (0.88, 1.29)
		45-54	0.86 (0.68, 1.09)	1.23 (0.98, 1.56)
		>=55	0.58 (0.48, 0.69)	1.16 (0.97, 1.38)
	F	<=24	1.18 (1.04, 1.33)	1.18 (1.04, 1.34)
		25-34	1.33 (1.15, 1.55)	1.03 (0.88, 1.19)
		35-44	1.27 (1.12, 1.43)	1.06 (0.93, 1.20)
		45-54	0.84 (0.71, 0.98)	1.05 (0.89, 1.23)
		>=55	0.77 (0.69, 0.86)	1.10 (0.98, 1.23)

© 2018 Sullivan SA et al. *JAMA Network Open.*

eTable 5. PPVs and 95% CIs of Pairs of Prodromal Symptoms

Symptom	ADHD-like	Bizarre behaviour	Blunted affect	Cannabis problems	Depressive symptoms	Role functioning problems	Social isolation	Symptoms of mania	OCD-like	Sleep disturbance	Smoking problems	Suicidal behaviour
ADHD-like	11.6 (10.9, 15.7)											
Bizarre behaviour	-	30.4										
Blunted affect	-	-	12.6 (7.3, 28.7)									
Cannabis problems	-	-	-	23.9 (22.2, 44.4)								
Depressive symptoms	14.5 (13.8, 20.9)	-	-	-	7.3 (7.7, 8.0)							
Role functioning problems	-	-	-	-	-	9.4 (8.2, 13.2)						
Social isolation	-	-	-	-	13.6 (11.3, 21.9)	-	11.5 (9.9, 17.1)					
Symptoms of mania	12.4 (11.4, 17.5)	-	-	-	6.1 (6.2, 6.7)	-	14.9 (10.7, 28.7)	6.1 (6.3, 6.8)				
OCD-like	-	-	-	-	13.9 (12.2, 21.2)	-	-	16.2 (12.9, 28.7)	11.8 (10.7, 16.7)			
Sleep disturbance	17.6 (16.3, 28.0)	-	-	-	4.5 (4.4, 5.1)	-	14.6	4.5 (4.4, 5.1)	18.6	4.5 (4.4, 5.1)		
Smoking problems	14.7 (12.7, 23.6)	-	-	-	8.6 (9.1, 9.8)	-	14.7 (10.4, 28.9)	8.3 (8.2, 9.9)	10.9 (7.8, 19.3)	7.6 (7.1, 9.6)	3.0 (2.9, 18.7)	
Suicidal behaviour	29.5 (24.2, 72.3)	-	-	-	26.8 (21.8, 42.1)	-	26.1	30.7 (24.7, 56.3)	43.9	29.1 (28.7, 58.6)	24.4 (26.2, 39.6)	24.1 (18.0, 36.2)

© 2018 Sullivan SA et al. *JAMA Network Open.*

Diagonal is PPV as a single symptom and row below are pairs of symptoms in the context of a background risk of 2.0%. PPVs have only been calculated when at least 10 cases had the symptom or pairs of symptoms (i.e. personal hygiene PPVs have not been calculated). 95% CI have not been calculated when any cell in the table was below 10. Numbers on diagonal are for single symptoms. Number in columns are for pairs of symptoms.

© 2018 Sullivan SA et al. *JAMA Network Open.*

eTable 6. Sensitivity Analyses: Association Between Prodromal Symptoms and Case-Control Status

Using only prodromal symptoms reported more than 1 year and more than 6 months before diagnosis

Prodromal symptom	Unadjusted OR	95% CI	p	Symptoms reported > 1 year before diagnosis OR	95% CI	p	Symptoms reported > 6 months before diagnosis OR	95% CI	p
ADHD-like symptoms	7.24	5.98, 8.77	≤0.001	3.40	2.65, 4.38	≤0.001	3.61	2.87, 4.57	≤0.001
Bizarre behaviour	21.89	8.01, 59.82	≤0.001	9.96	2.80, 35.44	≤0.001	11.69	3.41, 40.06	≤0.001
Blunted affect	7.59	3.79, 15.23	≤0.001	3.18	1.21, 8.36	0.019	3.91	1.64, 9.34	≤0.001
Cannabis related problems	15.87	11.19, 22.50	≤0.001	9.59	6.24, 14.76	≤0.001	10.05	6.70, 15.07	≤0.001
Depressive symptoms	12.14	11.56, 12.75	≤0.001	4.18	3.99, 4.38	≤0.001	4.55	4.36, 4.76	≤0.001
Role functioning problems	5.65	4.43, 7.21	≤0.001	3.23	2.33, 4.48	≤0.001	3.80	2.84, 5.08	≤0.001
Social isolation	6.62	5.03, 8.70	≤0.001	5.91	4.29, 8.15	≤0.001	6.13	4.54, 8.28	≤0.001
Mania-like symptoms	4.66	4.40, 4.94	≤0.001	3.17	2.97, 3.39	≤0.001	3.41	3.20, 3.63	≤0.001
OCD-like symptoms	6.89	5.48, 8.66	≤0.001	4.97	3.76, 6.59	≤0.001	5.24	4.03, 6.81	≤0.001
Personal hygiene	2.48	0.63, 9.79	0.194	1.39	0.15, 12.71	0.772	1.00	0.12, 8.45	1.000
Sleep disturbance	3.24	2.95, 3.56	≤0.001	2.27	2.03, 2.54	≤0.001	2.41	2.17, 2.67	≤0.001
Smoking related problems	2.00	1.91, 2.10	≤0.001	1.83	1.73, 1.93	≤0.001	1.88	1.78, 1.98	≤0.001
Suicidal behaviour	19.12	16.61, 22.02	≤0.001	8.79	7.37, 10.48	≤0.001	9.54	8.11, 11.23	≤0.001

© 2018 Sullivan SA et al. *JAMA Network Open.*

eFigure 1. Sensitivity Statistics for Prodromal Symptoms by Age Group and Sex

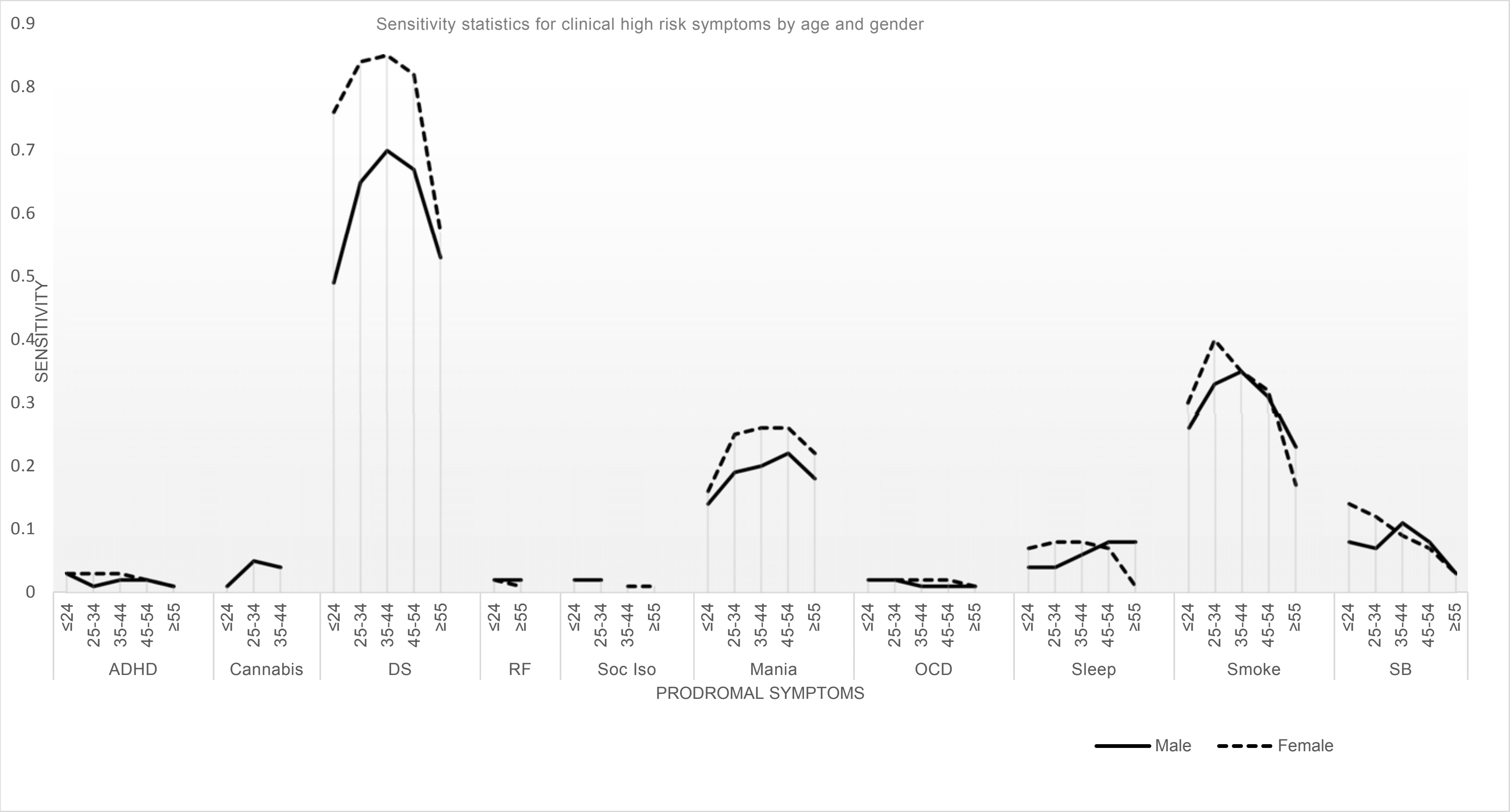

Key: ADHD – ADHD-like symptoms; Cannabis – problems with cannabis; DS – depressive symptoms; RF – role functioning problems; Soc Iso – social isolation; Mania – mania-like symptoms; OCD – OCD-like symptoms; Sleep – sleep disturbance; Smoke – problems associated with smoking tobacco; SB – suicidal behaviour

© 2018 Sullivan SA et al. *JAMA Network Open.*

eFigure 2. Forest Plot of Effect Sizes With Associated 95% CIs

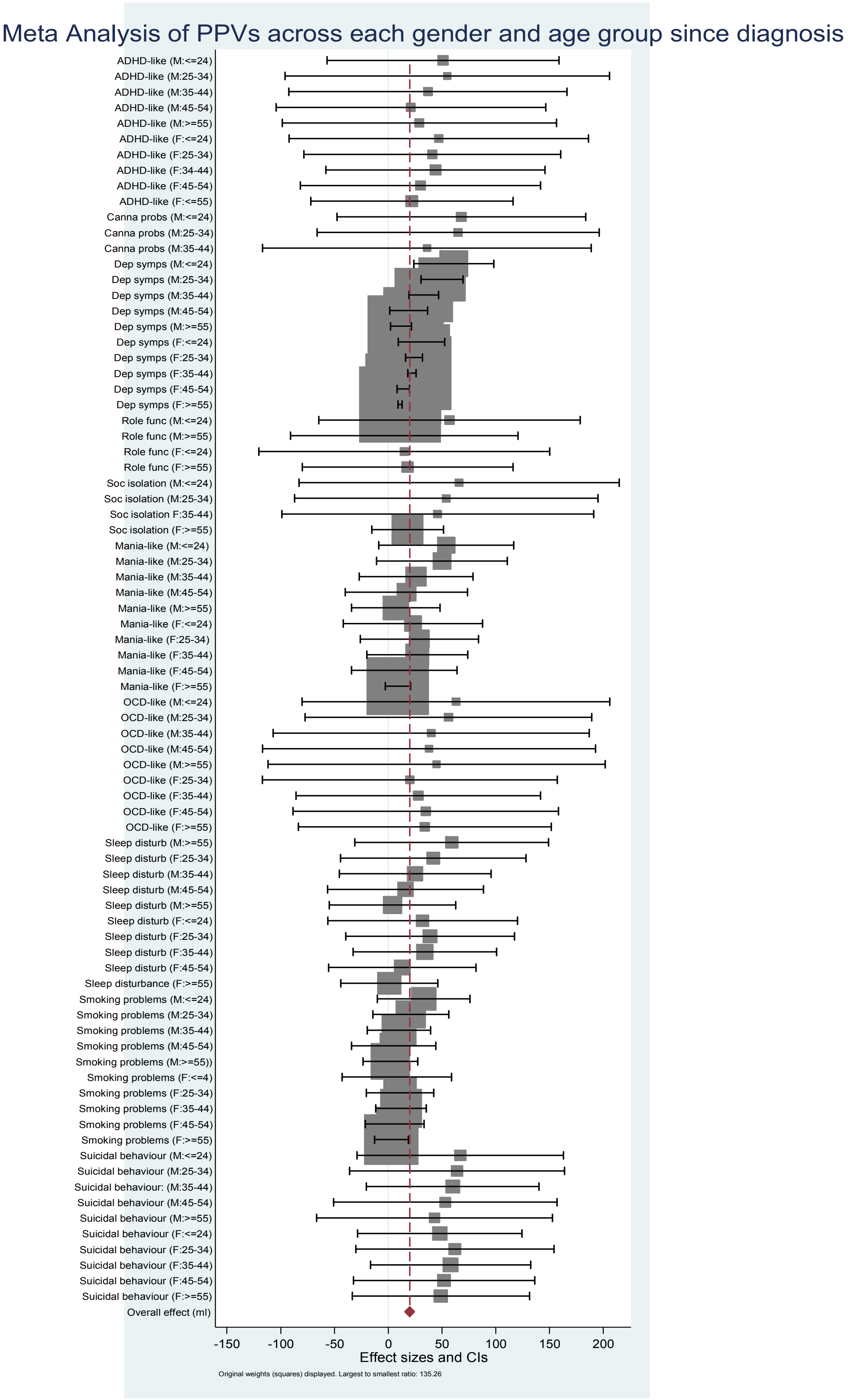

© 2018 Sullivan SA et al. *JAMA Network Open.*

© 2018 Sullivan SA et al. *JAMA Network Open.*
